# Hydrophobicity Modulation of Rose Bengal Derivatives in Homogeneous and Biomimetic Systems: Implications for Photodynamic Therapy

**DOI:** 10.1021/acsomega.6c07700

**Published:** 2026-10-10

**Authors:** Luiza Bertoldo Stefanello, Yara Shuvisnki Ricken, Miguel Sampaio, Carlos Vinícius Doerner dos Santos, Camila Fabiano de Freitas Marin

**Affiliations:** Department of Chemistry, 28117 Federal University of Santa Catarina (UFSC), Florianópolis, Santa Catarina 88040-900, Brazil

## Abstract

Rose Bengal (RB) is a highly efficient xanthene photosensitizer (PS) for photodynamic therapy (PDT). However, its high hydrophilicity and dianionic charge limit its interaction with biological membranes. To overcome these limitations, we synthesized octyl- (RBOCT) and dodecyl-substituted (RBDOD) RB esters to modulate hydrophobicity while preserving the photoactive xanthene core. Solvatochromic studies based on the ET(30) polarity scale and aggregation analyses in homogeneous media demonstrated that esterification increased lipophilicity (log*K*_p_ = 0.62, 1.65, and 2.27 for RB, RBOCT, and RBDOD, respectively), induced bathochromic shifts, enhanced fluorescence in less polar environments, and promoted aggregation in aqueous media, particularly for RBDOD, without compromising singlet oxygen generation in organic solvents. RB and its derivatives were also incorporated efficiently into biomimetic micellar systems composed of SDS, CTAB, TX-100, and SB3-14, with encapsulation efficiencies above 90%, appropriate hydrodynamic diameters (6.3–17.2 nm), and low polydispersity (0.21 < PDI < 0.46). Apparent p*K*_a_ analyses in micellar systems showed that RB predominantly exists as a dianion at physiological pH, whereas the ester derivatives remain mainly monoanionic. Micellar incorporation efficiently suppressed aggregation, restored the monomeric spectral features, significantly enhanced fluorescence quantum yields, and preserved the ability of all PSs to generate singlet oxygen. TX-100 and SB3-14 provided the most favorable microenvironments for fluorescence recovery. Stern–Volmer quenching studies demonstrated that micellar charge and polarity govern PS localization and protolytic equilibria, while increasing alkyl chain length promotes deeper insertion into less polar micellar domains. Overall, modulating molecular hydrophobicity and biomimetic microenvironments tailors the photophysical behavior of RB derivatives, providing design principles for optimized PSs and nanocarrier-based PDT formulations.

## Graphical abstract

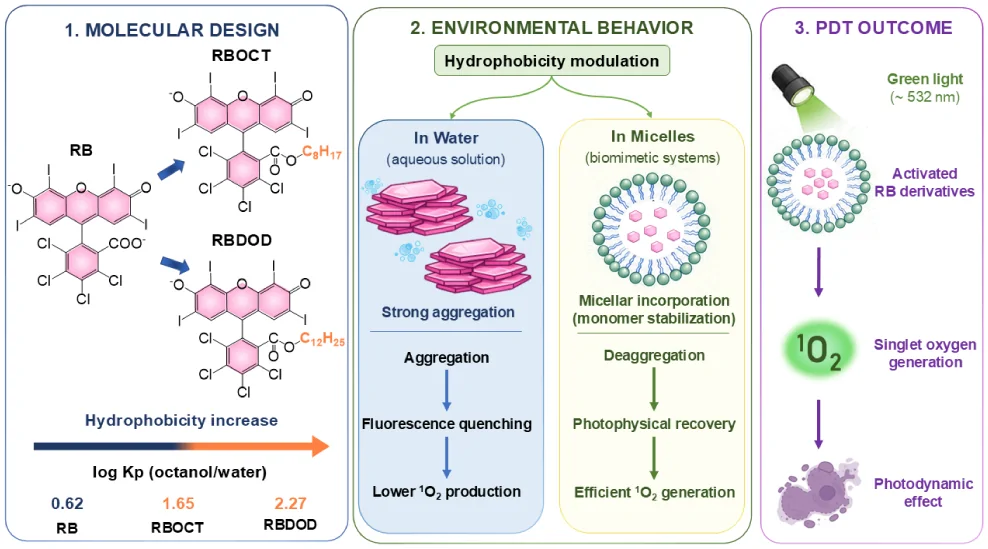

## 1 Introduction

Photodynamic therapy (PDT) is a noninvasive treatment modality for several diseases, including cancer, psoriasis, acne vulgaris, and microbial infections.^1,2^ Compared to conventional therapies, PDT offers important advantages, such as high spatial selectivity, the possibility of repeated treatments, and generally minimal systemic side effects.^3^ Its mechanism of action is based on the excitation of a photosensitizer (PS) by light at an appropriate wavelength in the presence of molecular oxygen, leading to the formation of cytotoxic reactive oxygen species (ROS), particularly singlet oxygen (^1^O_2_).^4^ Since ^1^O_2_ has a short lifetime, on the order of a few microseconds, and a diffusion radius of only a few hundred nanometers in aqueous media and cellular environments, the photodynamic effect is highly localized, making the spatial location of the PS within biological structures a decisive factor for therapeutic efficacy.^5^

The efficacy of PDT is therefore intrinsically related to the physicochemical and photophysical properties of the PS, which determine light absorption, excited-state dynamics, and ROS generation.^4,6^ To be considered suitable for PDT applications, a compound must have low dark toxicity, high triplet-state and singlet-oxygen quantum yields, adequate photostability, and sufficient solubility under physiological conditions.^4^ In addition to these intrinsic properties, the interaction of PSs with biomembranes and interfacial microenvironments plays a central role in determining their biological performance.^7^ In this context, the hydrophobicity and charge of the PS strongly influence membrane affinity, cellular uptake, and subcellular localization, thereby modulating photodynamic outcomes.^8^ However, achieving an optimal balance between aqueous solubility and membrane association remains a major challenge in the development of efficient PSs for PDT.^9,10^

Among the different classes of PSs investigated for PDT, xanthene dyes have attracted considerable attention due to their favorable photophysical properties, including strong absorption in the visible region, efficient intersystem crossing, and high ^1^O_2_ quantum yields.^11,12^ Within this family, Rose Bengal (RB) stands out because its chromophoric ring contains multiple heavy halogen substituents, which increase spin–orbit coupling and promote efficient population of the triplet excited state.^4,10^ RB exhibits intense absorption in the green spectral region (*ε* ≈ 9.5 × 10^4^ L mol^–1^ cm^–1^ near 550 nm in water) and reported singlet oxygen quantum yields of up to Φ_Δ_ ≈ 0.75 in aqueous media, along with established clinical use and low cost, making it an attractive PS for PDT and related applications.^10^ At physiological pH, RB exists predominantly in its dianionic form and exhibits excellent photophysical performance in homogeneous media, characterized by efficient triplet-state formation and effective ^1^O_2_ generation.^10^

Despite these advantages, the high hydrophilicity and dianionic character of RB significantly limit its interaction and penetration into phospholipid-based biological membranes, which can hinder membrane targeting and reduce PDT efficacy.^13,14^ To overcome these limitations, the structural modification of RB has emerged as a promising strategy. In particular, esterification of the benzoic carboxylate group reduces the overall negative charge and increases hydrophobicity while preserving the photoactive xanthene core^10,15^ (Figure 1a). Early studies on RB derivatives showed that structural modifications can generate less polar derivatives and substantially alter their spectral and photochemical properties.^16,17^ More recent studies on alkyl ester derivatives of xanthene dyes, including RB, eosin, and erythrosine, have demonstrated that such modifications can enhance affinity for biomimetic membranes and alter interfacial behavior without significantly compromising their intrinsic photodynamic properties when aggregation is controlled.^10,13,15,18,19^

**Figure 1:**
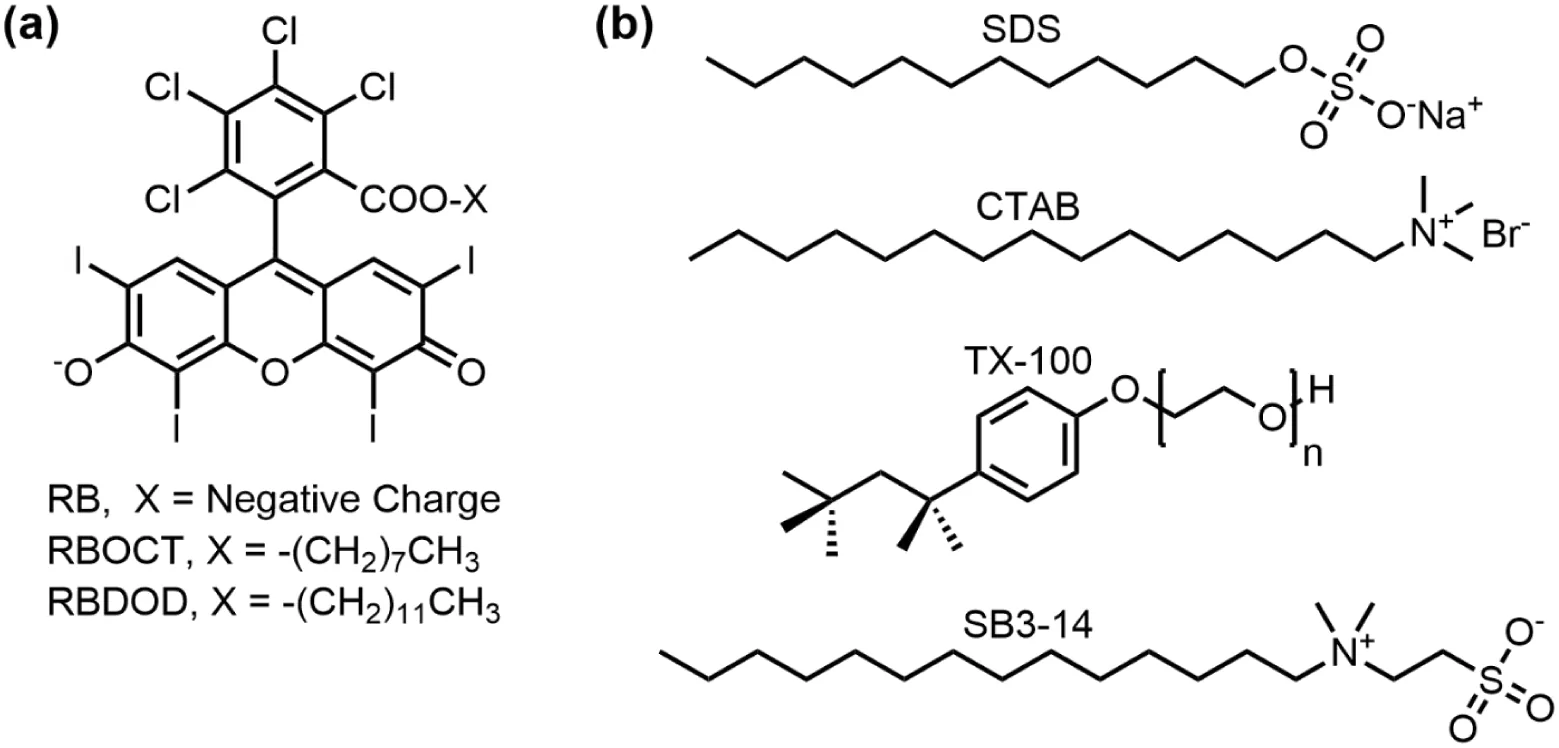
(a) Chemical structures of RB and its alkyl ester derivatives, Rose Bengal Octyl Ester (RBOCT) and Rose Bengal Dodecyl Ester (RBDOD). (b) Molecular structures of surfactants used to generate biomimetic micellar systems: SDS, CTAB, TX-100, and SB3-14.

Although several studies have reported the synthesis and biological potential of RB ester derivatives, a systematic understanding of how increasing alkyl chain length modulates their photophysical behavior, aggregation propensity, acid–base properties, and interactions with biomimetic interfaces remains limited.^10,15,20^ In particular, the combined influence of the solvent environment, hydrophobicity, and interfacial organization on the photoactive properties of RB derivatives has not been comprehensively investigated.

The interaction of photoactive drugs with biological membranes is governed by the molecular charge, hydrophobicity, and the physicochemical properties of the membrane surface.^8^ Electrostatic, hydrophobic, and hydration effects at a given pH strongly influence the affinity of PSs for lipid interfaces and their subsequent localization within cellular structures.^21^ In mammalian systems, plasma membranes are predominantly composed of zwitterionic phospholipids but may exhibit locally altered surface potentials.^22^ Tumor tissues often have increased negative surface charges associated with increased glycolytic activity and lactate secretion, while microbial envelopes, including those of many bacteria and fungi, are predominantly negatively charged.^23^ These variations critically affect PS-membrane interactions and, consequently, photodynamic performance in oncological and antimicrobial contexts.^24,25^

Since photodynamic action is highly localized and depends on the spatial proximity between the PS and its biological target, understanding how molecular charge and hydrophobicity modulate interfacial interactions is essential for predicting photodynamic efficiency.^5,24^ However, direct investigation of these processes in native membranes is hampered by their structural complexity, compositional heterogeneity, and dynamic behavior. As a result, simplified biomimetic systems have been widely employed to isolate and systematically evaluate the physicochemical parameters governing drug-membrane interactions at the interface level.^10,13,26^

Among these models, aqueous micelles represent a particularly useful class of biomimetic systems. Micelles consist of a hydrophobic core surrounded by a hydrated interfacial region, providing distinct microdomains that emulate key characteristics of biological membrane interfaces.^27^ In this context, micelles formed by surfactants with different headgroups offer direct membrane-mimetic environments (structures are shown in Figure 1b): anionic sodium dodecyl sulfate (SDS) represents highly hydrated and negatively charged interfaces, cationic cetyltrimethylammonium bromide (CTAB) probes electrostatically attractive environments for anionic PSs, nonionic Triton X-100 (TX-100) provides weakly hydrated and predominantly hydrophobic microdomains, and zwitterionic sulfobetaine SB3-14 more closely mimics the nearly neutral charge and interfacial organization of phosphatidylcholine-rich membranes.^22,26^ Together, these systems enable a systematic evaluation of how interfacial charge and hydration govern PS localization and photophysical response.

In this context, the present study investigates the interactions, localization, and photophysical behavior of RB and its alkyl ester derivatives in homogeneous media of different polarities and in aqueous microheterogeneous micellar environments. The analysis in homogeneous solvents establishes intrinsic photophysical trends and solvatochromic effects, providing a reference for interpreting the changes induced by interfacial confinement in the micellar systems. By correlating the molecular structure with micellar properties, this work aims to provide mechanistic insight into how hydrophobicity-driven partitioning and electrostatic interactions govern PS behavior at biomimetic interfaces relevant to photodynamic and antimicrobial applications. Such micellar models complement more complex lipid-based systems, emphasizing interfacial organization and allowing for rapid and well-defined screening of PS-interface interactions.

## 2 Methodology

### 2.1 Materials and Reagents

Rose Bengal (RB) was obtained from Nuclear (Brazil). 1-bromooctane, 1-bromododecane, sodium dodecyl sulfate (SDS, C_12_H_25_O_4_SNa, MW = 288.4 g mol^– 1^), cetyltrimethylammonium bromide (CTAB, C_19_H_42_NBr, MW = 364.5 g mol^– 1^), Triton X-100 (TX-100, (C_14_H_22_O­(C_2_H_4_O)_*n*_, MW = 647.0 g mol^– 1^) and *N*-tetradecyl-*N*,*N*-dimethyl-3-ammonio-1-propanesulfonate (SB3–14, C_19_H_41_NO_3_S, MW = 363.6 g mol^– 1^), as well as citric acid, disodium hydrogen phosphate (Na_2_HPO_4_), sodium iodide, phosphate-buffered saline (PBS), and alcohols (ethanol, 1-propanol, 1-pentanol, and 1-octanol), were purchased from Sigma-Aldrich (USA). Dimethylformamide (DMF), methanol, and hydrochloric acid (HCl) were obtained from Vetec (Brazil), whereas dimethyl sulfoxide (DMSO), ethyl acetate, and n-butanol were purchased from Neon (Brazil). All solvents were of analytical grade and used without further purification. Ultrapure water was obtained by reverse osmosis and used in all preparations.

### 2.2 Synthesis and Characterization of RBOCT and RBDOD

RB ester derivatives with 8- and 12-carbon alkyl chains, designated Rose Bengal Octyl Ester (RBOCT) and Rose Bengal Dodecyl Ester (RBDOD), were synthesized according to previously reported procedures.^15,20^ Reaction products were purified by silica gel column chromatography using ethyl acetate:ethanol (90:10, v/v) as the eluent. Structural characterization was performed by infrared spectroscopy (IR), proton nuclear magnetic resonance (^1^H NMR), carbon-13 nuclear magnetic resonance (^13^C NMR), and mass spectrometry (MS).

The purities of RBOCT and RBDOD were determined by quantitative ^1^H NMR (qNMR) using maleic acid as an internal standard, yielding purities of 91.5% and 97.1%, respectively. Detailed qNMR methodology, experimental conditions, spectra, integration data, and calculations are provided in the Supporting Information.

### 2.3 Photophysical Characterization in Homogeneous Media

#### 2.3.1 Sample Preparation

Stock solutions of RB, RBOCT, and RBDOD were prepared in DMSO at a concentration of 1.0 × 10^–3^ mol L^–1^ and subsequently diluted to the desired concentration for each experiment. All spectroscopic measurements were performed in quartz cuvettes with a 1.0 cm optical path length.

#### 2.3.2 UV–Vis Absorption Spectroscopy

UV–Vis absorption spectra were recorded in the 700–400 nm range using a Cary 50 spectrophotometer (Varian, USA). For all measurements, PSs concentration was fixed at 5 × 10^–6^ mol L^–1^. Apparent molar absorptivity (*ε*, L mol^–1^ cm^–1^) was determined from calibration curves obtained by plotting absorbance at the *λ*_max_ as a function of concentration (0.5–20 × 10^–6^ mol L^–1^), according to the Beer–Lambert law (*A* = *ε*cl). The final DMSO content was maintained below 1% (v/v). All measurements were performed in triplicate at room temperature.

#### 2.3.3 Fluorescence Spectroscopy and Quantum Yields

Fluorescence emission spectra were acquired using a Cary Eclipse spectrofluorometer (Varian, USA) with excitation wavelengths of 490 nm for RB and 520 nm for RBOCT and RBDOD. PSs concentration was fixed at 5 × 10^–7^ mol L^–1^. Fluorescence quantum yields (*Φ*_*F*_) were determined using an integrating sphere system (C9920-02, Hamamatsu Photonics, Japan) equipped with a photomultiplier tube (PMA-12, model C10027-02) and a xenon lamp as excitation source, under identical experimental conditions.

#### 2.3.4 Aggregation Studies in Water/Ethanol Mixtures

The aggregation behavior of RBOCT and RBDOD was investigated in binary ethanol–water mixtures (0–100% v/v). Final PSs concentrations were 5 × 10^–6^ mol L^–1^ for absorption and 5 × 10^–7^ mol L^–1^ for fluorescence measurements. Spectra were recorded at two time points: (i) 5 min after sample preparation and (ii) after 24 h of storage in the dark at room temperature, allowing evaluation of both rapid aggregation processes and potential time-dependent evolution.^28^

#### 2.3.5 Determination of the Partition Coefficient

Partition coefficients (*K*_*p*_) were determined using a biphasic water/1-octanol system at 1:1 (v/v). The aqueous phase consisted of PBS buffer at pH 7.4. Solutions containing 5 × 10^–6^ mol L^–1^ of each PS were prepared by adding concentrated DMSO stock solutions to the 1-octanol phase. The final DMSO content in the biphasic system was kept below 1% (v/v). The two phases were then vigorously mixed and allowed to equilibrate in the dark at room temperature for 48 h. After phase separation, the PS concentrations in both phases were determined by UV–Vis spectroscopy. The pH of the aqueous phase was not measured after equilibration. *K*_p_ values were calculated according to eq 1:

1 $$K_{p}=\frac{{[D]}_{octOH}}{{[D]}_{\mathrm{water}}}$$

#### 2.3.6 Degradation Kinetics of DPBF: Singlet Oxygen Quantum Yield

Singlet oxygen generation was evaluated using 1,3-diphenylisobenzofuran (DPBF) as a chemical probe.^29^ The reaction of DPBF with ^1^O_2_ leads to the formation of an endoperoxide, accompanied by the disruption of its extended π-electron system and the consequent loss of its characteristic spectroscopic features. This intermediate subsequently decomposes to yield 1,2-dibenzoylbenzene.

A stock solution of DPBF was prepared in DMSO and added to the sample to obtain a final concentration of 28 × 10^–6^ mol L^–1^. The samples containing RB and its derivatives in different solvents were irradiated using a green light source (λ_max_ = 516 nm) with an irradiance of 24 mW cm^–2^ for 60 min. DPBF consumption was monitored by measuring the decrease in its characteristic absorbance band at 420 nm with a UV–Vis spectrophotometer. Absorption spectra were collected at predetermined time intervals during irradiation. For each medium, control experiments were additionally performed to distinguish photosensitized DPBF consumption from probe instability. The kinetics of DPBF in the presence of each photosensitizer (DPBF + PS) were monitored under identical experimental conditions in the absence of irradiation (dark control) to verify that no significant DPBF consumption occurred without light. In parallel, DPBF photolysis was evaluated in the absence of PS (DPBF + light) using the same solvent, irradiation conditions, and acquisition intervals employed for the corresponding photosensitized experiment.

The rate of singlet oxygen production was determined from the decay of the DPBF absorbance at 420 nm, and a first-order kinetic equation was fitted to the experimental data, obtaining the rate constant for this reaction (*k*_DPBF_). The observed pseudo-first-order rate constant was obtained from eq 2, where *A*_0_ and *A*_t_ are the DPBF absorbances at 420 nm at times 0 and *t*, respectively:

2 $$\ln\left(A_{0}/A_{t}\right)=k_{\mathrm{obs}}\cdot t$$

To account for the direct photolysis of DPBF, the rate constant used for the singlet oxygen calculations was corrected by subtracting the rate constant measured for DPBF photolysis (*k*_photolysis(DPBF)_) in the corresponding solvent, according to eq 3:

3 $$k_{\mathrm{DPBF}}=k_{\mathrm{obs}}\left(PS+DPBF\right)-k_{\mathrm{photolysis}}\left(\mathrm{DPBF}\right)$$

The dark control kinetics were used as a stability control and were not included as an additional subtraction term when no significant DPBF decay was observed in the absence of irradiation.

To calculate the singlet oxygen quantum yield (Φ_Δ_), a series of equations were needed as described by Rabello.^30^ To determine the number of photons absorbed during the irradiation time (*t*) by the PS, eq 4 was utilized:

4 $$N_{\mathrm{ABS}}=\frac{1}{hcN_{a}}\int_{0}^{t}\int_{\lambda_{i}}^{\lambda_{f}}P_{e}\left(\lambda \right)\left(1-10^{-\mathrm{Abs}\left(\lambda \right)}\right)e^{-k_{\mathrm{PB}}t}\lambda d\lambda dt$$

Where *h* is the Planck constant, *c* is the speed of light, *N*_a_ is the Avogadro number, *P*_e_(*λ*) is the spectral power in (W cm^–1^), and λ_f_ and λ_i_ are the final and initial wavelengths of the integration interval, respectively. The chemical photodynamic efficiency (γΔ) was then calculated from the photolysis-corrected *k*_DPBF_ and *N*_ABS_ using eq 5:

5 $$\gamma_{\Delta }=\frac{k_{DPBF}}{N_{ABS}}$$

The singlet oxygen quantum yield (Φ_Δ_) of each PS was obtained by the comparative method according to eq 6, where $\Phi_{\Delta }^{\mathrm{Standard}}$ and $\gamma_{\Delta }^{\mathrm{Standard}}$ are the literature Φ_Δ_ and the experimentally determined photodynamic efficiency of the selected RB reference, respectively:

6 $$\Phi_{\Delta }=\frac{\Phi_{\Delta }^{\mathrm{Standard}}}{\gamma_{\Delta }^{\mathrm{Standard}}}{\gamma_{\Delta }}^{\mathrm{sample}}$$

Because both the lifetime/reactivity of ^1^O_2_ and the response of chemical trapping methods are solvent-dependent, RB measured in the same medium was preferentially used as the reference whenever a reliable literature Φ_Δ_ value was available. For aqueous medium and MeOH, Φ_Δ_ values of 0.75 and 0.76, respectively, were adopted from Murasecco-Suardi et al.^31^ For EtOH, Φ_Δ_ = 0.80 was adopted.^29^ For DMSO and DMF, Φ_Δ_ values of 0.16 and 0.47, respectively.^32^ For 1-propanol (PropOH), 1-butanol (ButOH), 1-pentanol (PentOH), and 1-octanol (OctOH), for which a suitable solvent-specific literature value for RB was not adopted, RB in EtOH was used as an external reference (Φ_Δ_ = 0.80). Consequently, the values obtained in these higher alcohols were treated as apparent singlet oxygen quantum yields (Φ_Δ,app_).

### 2.4 Characterization in Microheterogeneous Media

#### 2.4.1 Preparation of Micellar Media

Micellar solutions were prepared by dissolving each surfactant at 1% (w/v) in PBS buffer (pH 7.4), ensuring concentrations well above their respective critical micelle concentrations (CMCs). PSs were added from DMSO stock solutions and mixed to allow for incorporation into the micelles. The final DMSO content was kept below 1% (v/v) to preserve the micellar integrity. The physicochemical properties of the surfactants used in this study are summarized in Table 1.

**Table 1:** Physicochemical Properties of the Surfactants Used for Micelle Preparation

Surfactant	MW (g mol^–1^)	CMC (10^–3^ mol L^–1^) at 25 °C	*N*_agg_ at 25 °C	Solubility in water (g L^–1^) at 20 °C^a^
**SDS**	288.4	8.2 ^33^	62 ^33^	>130
**CTAB**	364.5	0.97 ^34^	90 ^35^	36.4
**TX-100**	647.0	0.22 ^36^	100 ^37^	Soluble
**SB3–14**	363.6	0.28 ^38^	70–76 ^39^	100

a Solubility data were obtained from Sigma-Aldrich safety data sheets, accessed in November 2025, for each surfactant.

#### 2.4.2 Dynamic Light Scattering (DLS) and Zeta Potential Measurements

Hydrodynamic diameter (*D*_h_), polydispersity index (PDI), and zeta potential (ζ) were measured using a Zetasizer Nanoseries instrument (Malvern, UK), equipped with a He–Ne laser (λ = 633 nm). Measurements were performed at 30 °C and a fixed scattering angle of 173°. Blank and PS-loaded micelles (5 × 10^–6^ mol L^–1^) were analyzed in triplicate.

#### 2.4.3 Micellar Encapsulation Efficiency (EE)

The total PS content (TC) in the micellar systems was determined after micelle dissolution in DMF. Briefly, 250 μL of the PS-loaded micelles (prepared at 1.0 × 10^–4^ mol L^–1^) were diluted to a final volume of 5 mL with DMF and stirred magnetically for 15 min at room temperature to ensure complete solubilization of the micellar aggregates. The PSs concentration in the resulting solution was quantified by UV–Vis spectroscopy, using calibration curves prepared in DMF over the range 0.5–20 × 10^–6^ mol L^–1^.

The amount of nonencapsulated (free PS; FC) was determined from the ultrafiltrate after ultrafiltration/centrifugation (Microcon 0.5 mL, 10,000 NMWL, Merck Millipore, Carrigtwohill, Ireland) carried out at 14,000 rpm for 30 min. The ultrafiltrate was dried under vacuum at 40 °C and then redissolved in DMF for quantification. Due to the lower concentration of PSs in the ultrafiltrate, the fluorescence spectroscopy method was employed. A separate calibration curve in DMF was used to ensure accurate quantification in this lower-concentration regime, with excitation at 490 nm for RB and 520 nm for RBOCT and RBDOD. Micellar encapsulation efficiency (EE) was calculated according to eq 7:

7 $$\mathrm{EE}\%=\frac{\mathrm{TC}-\mathrm{FC}}{\mathrm{TC}}x100$$

#### 2.4.4 Apparent p*K*_a_ Values in Micellar Media

The apparent p*K*_a_ values of the PSs in micellar media were determined by UV–Vis spectrophotometry over the pH range of 0.0–10.0. Solutions with pH values below 2.0 were prepared using standardized HCl, while the pH range from 2.0 to 8.0 was adjusted with McIlvaine buffer ([Na_2_HPO_4_] = [citric acid] = 7.5 × 10^–3^ mol L^–1^). For pH values between 8.0 and 10.0, a borate buffer ([H_3_BO_3_] = 7.5 × 10^–3^ mol L^–1^) was employed. A constant ionic strength was maintained by the addition of 0.10 mol L^–1^ NaCl as a background electrolyte. pH measurements were performed using an Ag/AgCl electrode with a 3.0 mol L^–1^ KCl reference electrolyte. It is important to note that, under strong alkaline conditions (pH > 12), RB undergoes rapid degradation due to hydrolytic cleavage of the pyran ring, a process favored by the presence of iodine substituents in the xanthene ring.^40^ For this reason, the pH range investigated in the present study was limited to 10.0.

A fixed aliquot of the PSs stock solution was added to each buffered system (approximately 40 experimental pH conditions), yielding a final PSs concentration of 5.0 × 10^–6^ mol L^–1^. The protonation equilibria were evaluated exclusively in the micellar phase (1% m/v surfactant), and the apparent p*K*_a_ values were obtained through multivariate analysis following methodologies previously applied to protolytic systems of xanthene dyes.^9,14,41^ Data processing was carried out using MASDA (Multivariate Analysis of Spectroscopic Data), in which the complete set of UV–Vis spectra recorded as a function of pH was analyzed simultaneously, rather than the absorbance variation at a single wavelength. Principal component analysis (PCA) was initially used to estimate the number of significant spectroscopically distinguishable components contributing to the experimental data set. The spectral data were subsequently subjected to Imbrie Q-mode factor analysis followed by Varimax rotation and Imbrie oblique projection to obtain the relative concentration profiles of the contributing species as a function of pH.^42^ The software was developed at the Laboratory of Chemometrics and Natural Sciences, State University of Londrina (Brazil).^43^

Furthermore, it is important to emphasize that the experimental p*K*_a_ values determined in this study are affected by the colloidal environment of the system. Therefore, these values are herein referred to as apparent p*K*_a_ values. Following the determination of the apparent p*K*_a_, distribution curves of the pure protolytic species were constructed for each pH condition by applying the relative species concentrations using the Henderson–Hasselbalch equation.

#### 2.4.5 Determination of Pure Protolytic Spectra and Molar Absorptivities

The pure spectra of the individual protolytic species were resolved using the K-matrix method, a multivariate spectral deconvolution approach based on the mathematical treatment of absorbance data obtained over the investigated pH range and implemented in the MATK1 module of MASDA. This method enables the extraction of pure spectra and the determination of the corresponding molar absorptivity coefficients for each protolytic species.

The tautomeric forms were not introduced as predefined spectral models but were assigned after multivariate resolution based on the recovered spectral characteristics, their pH-dependent relative concentration profiles, and the established acid–base and tautomeric behavior of xanthene dyes.^41^ No predefined spectra or specific spectral band-shape constraints were imposed during the multivariate analysis.

#### 2.4.6 UV–Vis Absorption, Fluorescence Emission, and Quantum Yields

UV–Vis absorption spectra were recorded on a Cary 60 spectrophotometer (Agilent), and fluorescence emission spectra were obtained on a Cary Eclipse spectrofluorometer under conditions analogous to those employed in homogeneous media. Φ_F_ values were determined using an integrating sphere, with concentrations adjusted to avoid inner-filter effects.

The singlet oxygen quantum yield was determined following the same procedure described for homogeneous media (Section 2.3.6). In micellar systems, DPBF was added 30 min before the measurements, and the samples were kept under continuous stirring in the dark to promote the incorporation of the probe into the micellar phase.

#### 2.4.7 Fluorescence Quenching Studies: Determination of the Stern–Volmer Constant

Fluorescence quenching experiments were carried out using iodide ions (I^–^) as the quencher. The PS solutions were prepared in the presence of different surfactants: TX-100 and SB3–14 at a concentration of 1.0% (w/v), and CTAB and SDS at 0.15% (w/v). Stock solutions of iodide were prepared in the corresponding surfactant media at a concentration of 1.00 mol L^–1^ for TX-100, SB3-14, and SDS systems, whereas a 0.10 mol L^–1^ iodide stock solution was used for CTAB due to precipitation observed at higher iodide concentrations. Aliquots of the iodide stock solutions were successively added to the photosensitizer solutions, and fluorescence emission spectra were recorded after each addition.

The Stern–Volmer quenching constant (*K*_SV_) was calculated from eq 8

8 $$\frac{F_{0}}{F}=1+k_{\mathrm{SV}}\left[I^{-}\right]$$

where *F*_0_ and *F* are the fluorescence intensities in the absence and presence of the quencher, respectively, and [I^–^] is the analytical iodide concentration added to the sample. Local interfacial iodide concentrations were not independently determined.

#### 2.4.8 Determination of Fluorescence and Singlet Oxygen Quantum Yields

The fluorescence quantum yields (Φ_F_) and singlet oxygen quantum yields (Φ_Δ_) of all compounds in the micellar systems were determined using the same methodology described in Sections 2.3.3 and 2.3.6, respectively. For the determination of Φ_Δ_, the DPBF probe was added to the micellar systems and allowed to equilibrate to ensure its incorporation into the micelles before irradiation. Only after this equilibration period was the irradiation procedure initiated. Control experiments were also performed under the same conditions to evaluate DPBF photolysis in each micellar medium and DPBF stability in the presence of the PSs in the absence of irradiation. The DPBF degradation rate constants (*k*_DPBF_) used for the calculations were corrected for the corresponding probe photolysis.

For the micellar systems (SDS, CTAB, TX-100, and SB3–14), RB in EtOH (Φ_Δ_ = 0.80)^29^ was used as an external reference, following the same comparative approach described in Section 2.3.6. EtOH was selected as a homogeneous medium in which RB and DPBF are molecularly dispersed and for which a well-established Φ_Δ_ value is available. Because DPBF reactivity and local concentration may be affected by compartmentalization in micellar environments, the values obtained in these systems were reported as apparent singlet oxygen quantum yields (Φ_Δ,app_) and interpreted as comparative indicators of photosensitizing efficiency.

## 3 Results and Discussion

### 3.1 Characterization of the Synthesized Ester Derivatives

The structural characterization of derivatives RBOCT and RBDOD was carried out by IR, NMR, and LRMS analyses, and the results obtained were compared with those reported in the literature.^15,17,20^

The IR spectra exhibited the characteristic ester C=O stretching band at 1734 cm^–1^ for RBOCT and 1737 cm^–1^ for RBDOD, in agreement with the typical range reported for ester carbonyl groups (1720–1760 cm^–1^).^20^ Also, the broad O–H band between 2700 cm^–1^ and 3300 cm^–1^ that was previously present in the starting material was no longer observed.

The ^1^H and ^13^C NMR spectra further confirmed the successful esterification. Signals associated with the incorporated alkyl chains were observed in the 0.84–1.31 ppm region (^1^H NMR) and 14.5–31.8 ppm (^13^C NMR). In addition, the signals observed at 3.91–3.93 ppm in both derivatives can be assigned to the methylene group directly bonded to the ester oxygen.

Finally, LRMS analysis revealed *m*/*z* peaks at 1084 (100%) for RBOCT and 1140 (100%) for RBDOD, consistent with the expected mass-to-charge ratios of the molecular ions for both compounds. All spectroscopic data and complete characterization are provided in the Supporting Information (Figures S1–S9).

### 3.2 Solvent-Dependent Properties in Homogeneous Media

#### 3.2.1 Steady-State Absorption and Emission Characteristics in Different Solvents

Xanthene dyes characteristically exhibit an intense absorption band assigned to the S_0_ → S_1_ transition, and a secondary shoulder corresponding to the S_0_ → S_2_ transition.^10^ The esterification of RB at its carboxylate moiety does not change these photophysical features, but it ultimately results in a bathochromic shift, ranging from 4 to 16 nm for RBOCT and 6 to 19 nm for RBDOD, depending on the solvent (Table 2). This behavior is typically attributed to modifications in the electronic environment surrounding the chromophore upon ester formation. In particular, the reduction of intramolecular hydrogen bonding and changes in excited-state stabilization can contribute to this observation.^9,10^

**Table 2:** Photophysical Parameters of RB, RBOCT, and RBDOD in Different Solvents^a^

PS	Solvent	*λ*_abs_ (nm)	*λ*_em_ (nm)	Δ*υ* (nm)	ε *ε* (10^3^ L mol^–1^cm^–1^)	Φ_F_
**RB**	Water	542	556	14	69.0 ± 0.2	0.014 ± 0.004
	MeOH	550	562	12	74.4 ± 2.9	0.091 ± 0.001
	EtOH	553	567	14	82.0 ± 0.6	0.061 ± 0.004
	PropOH	556	567	11	83.0 ± 0.4	0.035 ± 0.002
	ButOH	556	568	12	90.0 ± 1.2	0.046 ± 0.001
	PentOH	557	570	13	86.9 ± 1.0	0.045 ± 0.001
	OctOH	565	574	9	N.D.	0.007 ± 0.001
	DMSO	560	571	11	93.5 ± 1.0	0.107 ± 0.001
	DMF	558	572	14	105.6 ± 1.2	0.148 ± 0.003
**RBOCT**	Water	558	N.D.	N.D.	N.D.	0.013 ± 0.004
	MeOH	557	571	14	105.3 ± 0.9	0.112 ± 0.001
	EtOH	562	576	14	61.0 ± 0.7	0.114 ± 0.001
	PropOH	564	576	12	102.7 ± 2.4	0.136 ± 0.002
	ButOH	564	578	14	99.4 ± 1.4	0.153 ± 0.001
	PentOH	565	579	14	102.5 ± 1.6	0.164 ± 0.001
	OctOH	569	579	10	57.9 ± 1.4	0.123 ± 0.001
	DMSO	568	582	14	90.1 ± 0.6	0.348 ± 0.003
	DMF	567	581	14	116.9 ± 0.6	0.332 ± 0.001
**RBDOD**	Water	561	N.D.	N.D.	N.D.	0.010 ± 0.001
	MeOH	560	572	12	142.1 ± 1.2	0.134 ± 0.001
	EtOH	564	577	13	86.0 ± 0.6	0.163 ± 0.002
	PropOH	566	579	13	157.4 ± 3.6	0.172 ± 0.001
	ButOH	567	578	11	148.2 ± 1.3	0.190 ± 0.003
	PentOH	568	578	10	142.8 ± 1.1	0.189 ± 0.001
	OctOH	571	581	10	107.7 ± 0.8	0.178 ± 0.001
	DMSO	569	583	14	149.3 ± 1.1	0.341 ± 0.002
	DMF	568	583	15	151.7 ± 0.7	0.375 ± 0.001

a N.D., not detected under the experimental conditions.

In ethanol, for instance, RB exhibits a maximum absorption at 553 nm with an apparent molar absorptivity of 82 × 10^3^ L mol^–1^ cm^–1^, consistent with values reported in the literature.^44^ The ester derivatives show slightly red-shifted maxima (562 nm for RBOCT and 564 nm for RBDOD) while maintaining high *ε* values (Table 2). In less polar media, both derivatives display higher apparent molar absorptivities, consistent with improved solubility of the alkyl-substituted dyes and a reduced tendency to form aggregated species. This bathochromic shift, along with the maintenance of high absorptivity values, is particularly advantageous for photodynamic applications, as the use of longer wavelengths can improve light penetration in biological tissues.^45^ Since the xanthene core remains the main contributor to the electronic transitions, esterification at the carboxylate group does not substantially perturb the intrinsic photophysical behavior of the molecule as a whole.^10^ The normalized absorption and emission spectra of RB, RBOCT, and RBDOD recorded in different solvents are presented in Figure 2. For all three PSs, the overall spectral profiles are preserved across the solvent series. This observation confirms that chemical modification mainly modulates solvatochromic behavior rather than altering the fundamental electronic structure of the dyes.

**Figure 2:**
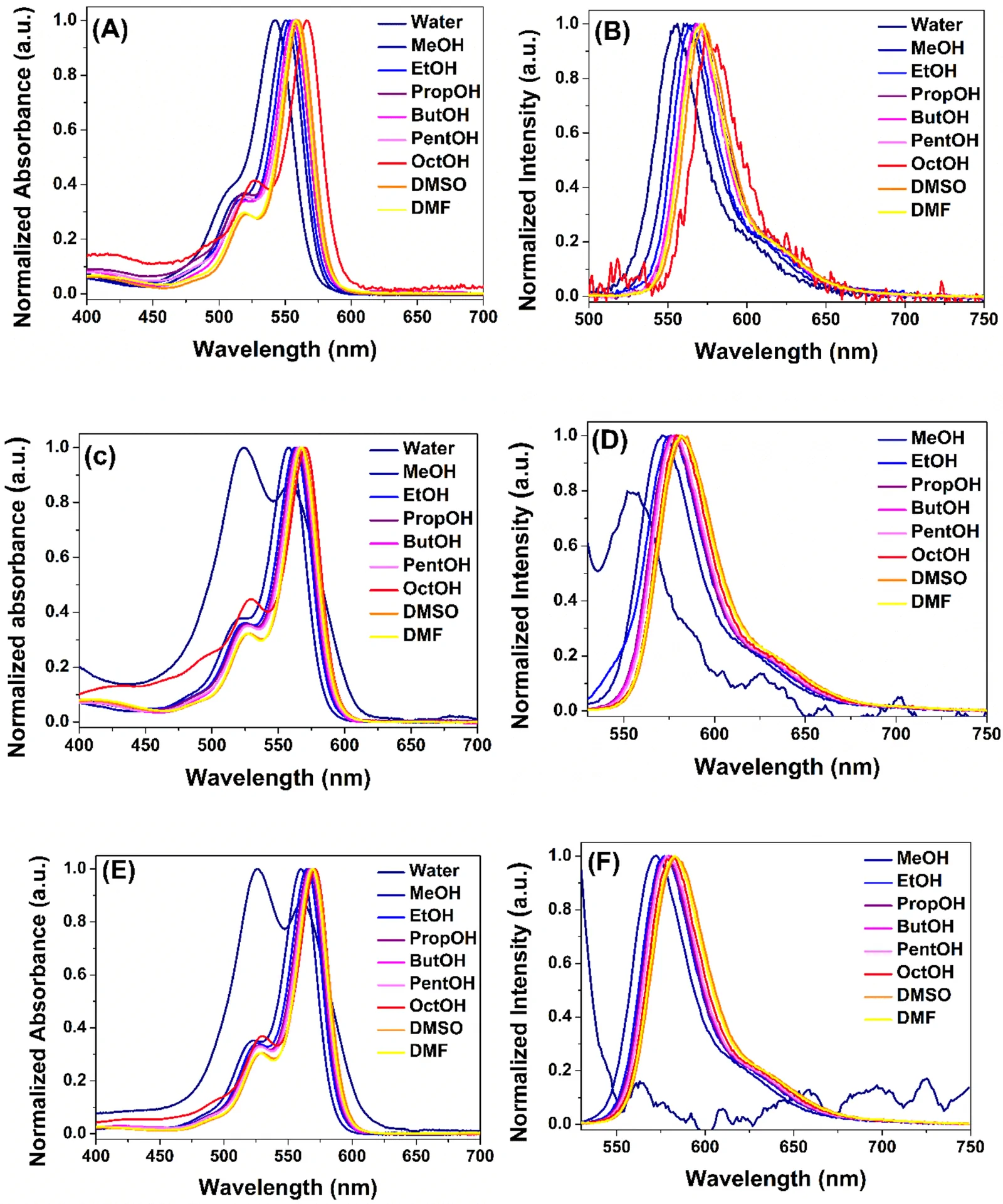
Normalized absorption (A, C, E) and emission (B, D, F) spectra of RB, RBOCT, and RBDOD in various solvents, respectively. All spectra were normalized to unity at the maximum peak (*A*/*A*_max_ or *I*/*I*_max_ = 1.0). In water media, the emission of RBOCT and RBDOD is very weak and appears as a noisy baseline due to extensive aggregation and quenching.

In an aqueous solution, pronounced differences arise due to the increased hydrophobicity of the ester derivatives. Both RBOCT and RBDOD exhibit spectral features qualitatively consistent with aggregation (Figure 2C,E), including a pronounced decrease in apparent molar absorptivity and the absence of detectable fluorescence emission (Figure 2D,F). These effects are largely reversed upon incorporation into micellar systems (see Section 3.3.1), indicating that aggregation processes dominate in water but are suppressed in organic media. Such behavior is expected, as longer alkyl chains enhance hydrophobic interactions and esterification reduces the ionic charge of the dye, conditions that favor aggregation in aqueous media. Similar aggregation-induced changes in optical properties have been reported for esterified analogues of RB,^10^ eosin,^13^ and erythrosine.^9,14^

Solvent polarity significantly influences the photophysical behavior of RB and its ester derivatives, consistent with the solvatochromic profiles typically observed for xanthene dyes.^46^ Across the protic solvent series, all three compounds exhibit a clear bathochromic shift as solvent polarity decreases (Table 2). For RB, *λ*_max_ increases from 542 nm in water to 565 nm in octanol. A similar shift occurs for RBOCT (558 → 569 nm) and RBDOD (561 → 571 nm). This positive solvatochromism reflects differences in the relative stabilization of the ground and excited states as a function of the solvent environment.^46,47^ As a result, the S_0_ → S_1_ energy gap is reduced, and the transition shifts to longer wavelengths. In agreement with these observations, several studies report that when RB is transferred from water into less polar media, such as alcohol–water mixtures or micelles, its maximum absorption shifts to a longer wavelength.^46^

To describe the effects of solvent polarity on the photophysical behavior of RB and its ester derivatives, the empirical parameter *E*_T_(30) was employed. This parameter was originally introduced by Reichardt based on the solvatochromic charge-transfer band of betaine-30. It reflects the combined influence of solvent polarity, polarizability, and specific solute–solvent interactions, particularly hydrogen-bonding ability.^48,49^ In the present work, *E*_T_(30) values were estimated for each PS–solvent pair from the absorption maximum according to eq 9, which converts the absorption wavelength into the corresponding electronic transition energy expressed in kcal mol^–1^.^50,51^

9 $$E_{T}\left(30\right)=\frac{28,591}{\lambda_{\max}^{\mathrm{abs}}}$$

The resulting *E*_T_(30) values (Table S1 and Supporting Information) were used to construct the correlation plot shown in Figure 3. A clear linear trend is observed across the entire series of alcohols for all three PSs, indicating a systematic dependence of the electronic transition energy on the solvent polarity within this class of protic solvents. Deviations from this linear trend are observed for polar aprotic solvents (DMF and DMSO), indicating that solvent effects on the photophysical behavior of xanthene dyes cannot be explained in terms of the dielectric constant or general polarity only. Although the dielectric constant is used here as a reference parameter, deviations observed for aprotic solvents highlight the dominant role of specific solute–solvent interactions, particularly hydrogen bonding and dipolar stabilization.^46,48^ Similar correlations between *E*_T_(30) values and solvatochromic shifts have been reported for related xanthene dyes, such as eosin, including comparisons with alternative polarity scales (e.g., Kosower Z), supporting the suitability of *E*_T_(30) to describe solvent effects in this chromophore family.^51^

**Figure 3:**
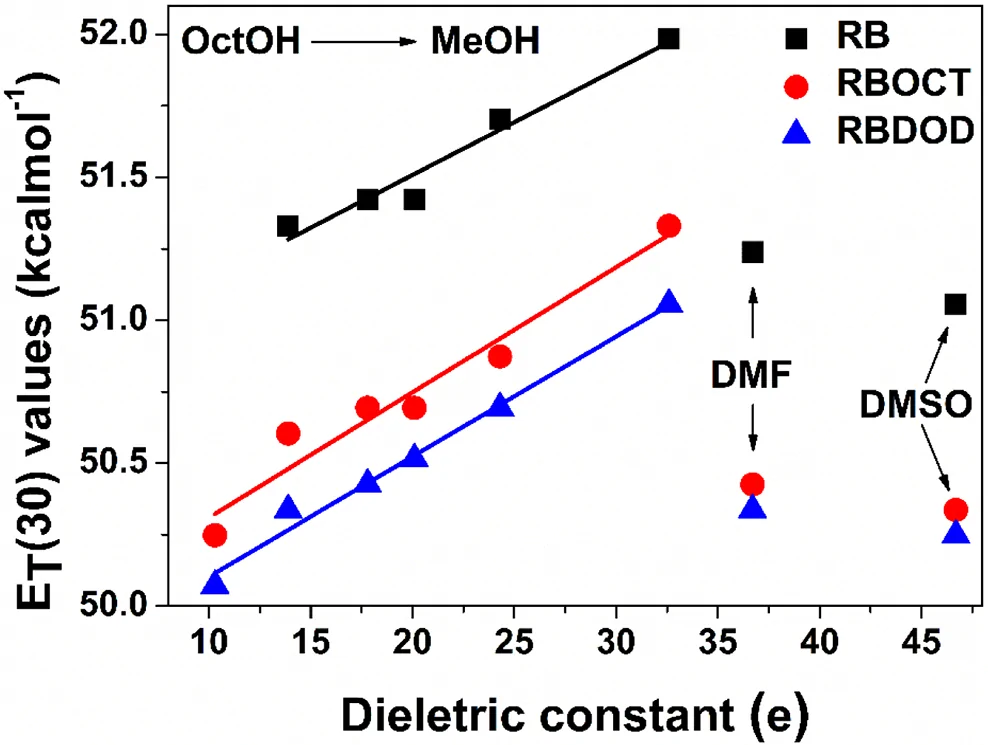
Correlation between E_T_(30) values of RB, RBOCT, and RBDOD and the dielectric constant of the solvents. Straight lines correspond to linear fits for the alcohol series (from octanol to methanol); polar aprotic solvents (DMF and DMSO) deviate from this trend.

In this context, the pronounced bathochromic shifts observed in polar aprotic solvents can be explained by the nature of their solvation mechanisms. Although DMF and DMSO have high dielectric constants, they do not act as hydrogen-bond donors but stabilize the chromophore mainly through dipole–dipole interactions, and they often reduce RB aggregation relative to water.^46^ In such solvents, the charge-transfer character of *S*_1_ is relatively better stabilized, which reduces the S_0_ → S_1_ energy gap and favors a bathochromic shift, as seen in Table 2. Studies following RB and related xanthene compounds in different solvents show that changes in solvent hydrogen-bonding ability systematically tune absorption maxima, consistent with the present findings.^46^

The fluorescence emission behavior follows trends like those observed in absorption (Figure 2). The emission maxima shift to longer wavelengths as solvent polarity decreases along the alcohol series. Polar aprotic solvents display emission bands at higher wavelengths compared to the alcohol series (except for octanol), which is consistent with stronger relative stabilization and more complete relaxation of the RB *S*_1_ state in these media.^46,47^ In octanol, both RB and its ester derivatives display a smaller Stokes shift compared to those of shorter-chain alcohols (Table 2). This behavior is likely due to the higher viscosity and more structured hydrogen-bond network of octanol.^52^ These properties restrict the full relaxation of the excited state before emission, causing the emission maximum to lie closer to the absorption maximum. Time-resolved studies on RB and similar xanthene dyes have shown that emission maxima and intersystem crossing rates are markedly modulated by solvent polarity, viscosity, and hydrogen-bonding ability, reflecting strong environmental control of the relaxed excited state.^46,53^

Fluorescence quantum yield $\left(\Phi_{F}\right)$ ­(Φ_F_) further reinforces the solvent dependence of excited-state processes. For the ester derivatives, Φ_F_ generally increases as solvent polarity decreases along the alcohol series, reaching maximum values in pentanol, followed by a slight decrease in octanol (Table 2). These variations are consistent with the combined effect of polarity, viscosity, and hydrogen-bonding ability.^54,55^ For all three PSs, Φ_F_ showed higher values in polar aprotic solvents, where nonradiative decay pathways such as hydrogen-bond-assisted deactivation, faster intersystem crossing, and aggregation are less effective.^46,47^ The higher Φ_F_ values of the derivatives compared with RB in most organic solvents indicate that alkyl esterification in these media reduces aggregation and the associated nonradiative decay pathways.^9,10,13^

The results of the DPBF degradation kinetics analysis used to determine the singlet oxygen quantum yield are presented in Table 3.

**Table 3:** Kinetic and Photophysical Parameters Obtained Through the Reaction between ^1^O_2_ and DPBF; [PS] = 5.0 × 10^–6^ mol L^–1^; pH 7.4 and 25.0 °C^f^

PS	Solvent	*K*_DPBF_ (10^–2^ s^–1^)	*P*_ABS_ (10^–3^W)	*N*_ABS_ (10^–4^E)	γΔ	φ_Δ_
**RB**	Water	0.17 ± 0.03	18.70 ± 0.11	2.92 ± 0.02	5.82 ± 1.03	0.75^b^
	MeOH	8.57 ± 0.95	7.58 ± 0.13	1.20 ± 0.02	714.17 ± 80.06	0.76^c^
	EtOH	0.15 ± 0.02	7.27 ± 0.01	1.15 ± 0.01	13.04 ± 1.74	0.80^a^
	PropOH	0.12 ± 0.02	7.02 ± 0.11	1.11 ± 0.02	10.81 ± 1.81	0.66^a^
	ButOH	0.12 ± 0.04	6.94 ± 0.16	1.10 ± 0.02	10.91 ± 3.64	0.67^a^
	PentOH	0.11 ± 0.03	6.05 ± 0.06	0.96 ± 0.01	11.46 ± 3.13	0.70^a^
	OctOH	1.99 ± 0.72	1.40 ± 0.06	0.22 ± 0.01	904.55 ± 329.85	55.48^a^
	DMSO	5.07 ± 0.18	6.11 ± 0.07	0.97 ± 0.02	522.68 ± 21.46	0.16^d^
	DMF	6.00 ± 1.65	18.70 ± 0.14	2.92 ± 0.02	205.48 ± 56.52	0.47^e^
**RBOCT**	Water	0.16 ± 0.26	18.40 ± 0.32	2.87 ± 0.05	5.57 ± 9.06	0.72^b^
	MeOH	8.43 ± 1.31	7.80 ± 0.06	1.24 ± 0.01	679.84 ± 105.79	0.72^c^
	EtOH	0.17 ± 0.01	7.05 ± 0.05	1.12 ± 0.01	15.18 ± 0.90	0.93^a^
	PropOH	0.14 ± 0.03	7.27 ± 0.44	1.17 ± 0.08	11.97 ± 2.69	0.73^a^
	ButOH	0.16 ± 0.03	6.92 ± 0.15	1.10 ± 0.02	14.55 ± 2.74	0.89^a^
	PentOH	0.13 ± 0.01	6.54 ± 0.08	1.04 ± 0.02	12.50 ± 0.99	0.77^a^
	OctOH	9.72 ± 0.07	5.90 ± 0.18	0.93 ± 0.03	1045.16 ± 34.54	64.10^a^
	DMSO	7.39 ± 0.33	5.74 ± 0.02	0.91 ± 0.01	812.09 ± 37.35	0.25^d^
	DMF	15.38 ± 0.45	18.70 ± 0.59	2.92 ± 0.09	526.71 ± 22.38	1.20^e^
**RBDOD**	Water	N.D.	-	-	N.D.	N.D.
	MeOH	9.63 ± 2.00	9.56 ± 0.11	1.51 ± 0.02	637.75 ± 132.72	0.68^c^
	EtOH	0.16 ± 0.02	8.71 ± 0.18	1.38 ± 0.02	11.59 ± 1.46	0.71^a^
	PropOH	0.15 ± 0.06	8.38 ± 0.02	1.33 ± 0.01	11.28 ± 4.51	0.69^a^
	ButOH	0.16 ± 0.04	8.43 ± 0.24	1.34 ± 0.03	11.94 ± 3.00	0.73^a^
	PentOH	0.19 ± 0.02	8.26 ± 0.04	1.31 ± 0.01	14.50 ± 1.53	0.89^a^
	OctOH	10.70 ± 0.52	7.59 ± 0.05	1.20 ± 0.01	891.67 ± 43.97	54.69^a^
	DMSO	6.44 ± 0.09	7.38 ± 0.11	1.17 ± 0.02	550.43 ± 12.15	0.17^d^
	DMF	17.08 ± 2.59	18.50 ± 0.20	2.88 ± 0.03	593.06 ± 90.14	1.36^e^

a RB in EtOH (ΦΔ = 0.80)^29^ was also used as an external reference for PropOH, ButOH, PentOH, and OctOH.

b RB in water/PBS (ΦΔ = 0.75)^31^.

c RB in MeOH (ΦΔ = 0.76)^31^.

d RB in DMSO (ΦΔ = 0.16)^32^.

e RB in DMF (ΦΔ = 0.47)^32^.

f N.D.: not detected under the experimental conditions.

Control experiments performed in the absence of irradiation showed no significant DPBF degradation, indicating that dark reactions were negligible under experimental conditions. Therefore, the *k*_DPBF_ values reported in Table 3 were obtained by subtracting the DPBF photolysis rate constant determined in the absence of the PS from the observed rate constant (*k*_obs_), according to eq 3.

The singlet oxygen quantum yields (Φ_Δ_) obtained for RB and its ester derivatives (Table 3) indicate that esterification retains the photodynamic competence of the xanthene chromophore in most organic media evaluated. In MeOH and EtOH, the derivatives showed Φ_Δ_ values comparable to RB, while in PropOH, ButOH, and PentOH they maintained high apparent singlet oxygen quantum yields (Φ_Δ,app_). For OctOH, however, normalization to RB in EtOH produced nonphysical Φ_Δ,app_ values (>1); therefore, these estimates were not considered quantitative measures of ^1^O_2_ generation. The unusually high DPBF consumption observed in OctOH likely reflects the strong dependence of the chemical probe response on the local solvent environment. In particular, the substantially higher viscosity of OctOH compared with shorter-chain alcohols may affect diffusion and the encounter dynamics between DPBF and ^1^O_2_. Since DPBF oxidation is sensitive to solvent-dependent factors, including probe reactivity, ^1^O_2_ lifetime, and mass transport, the response observed in OctOH cannot be attributed exclusively to enhanced ^1^O_2_ generation and precludes direct quantitative comparison with the EtOH reference.^29^ These results align with the solvatochromic behavior discussed above, where the ester derivatives exhibited systematic bathochromic shifts while preserving the characteristic spectral features of the RB chromophore. Overall, the data suggest that increasing hydrophobicity through esterification modifies the interaction of the dye with its environment without substantially compromising the excited-state pathways relevant to singlet oxygen generation in media that favor molecular dispersion and for which the comparative DPBF method provides physically meaningful values.^10,13,56^

The relationship between solvent properties and photodynamic efficiency is not, however, governed by polarity alone. Water provides the clearest example: RBOCT retained an apparent Φ_Δ_ of 0.72, close to that of RB (0.75), whereas ^1^O_2_ generation by RBDOD was not detected under the experimental conditions, consistent with the aggregation phenomena identified by absorption and fluorescence spectroscopy (Figure 2). In contrast, in media that favor predominantly monomeric species, the photosensitizing efficiency of the derivatives is largely recovered. In this sense, aggregation appears to be the main factor limiting photodynamic performance in aqueous environments.^9,10,13^

The comparison between RBOCT and RBDOD further shows that increasing alkyl chain length does not yield a simple monotonic change in singlet oxygen generation. Although both derivatives maintained substantial singlet oxygen generation in most organic solvents, neither displayed a consistent advantage across the entire solvent set. In DMSO, Φ_Δ_ values were 0.25 and 0.17 for RBOCT and RBDOD, respectively. In DMF, the comparative calculation yielded apparent values of 1.20 and 1.36, respectively; because these exceed the physical limit for the singlet-oxygen quantum yield, they were interpreted as evidence that the DPBF-based response in this medium cannot be translated directly into an absolute Φ_Δ_ using the adopted reference procedure. This solvent-dependent behavior agrees with previous studies on RB derivatives and related xanthene dyes, which demonstrate that photosensitizing efficiency is governed by a complex interplay between local solvation, molecular organization, and aggregation phenomena rather than by bulk polarity alone.^9,10,13,46^

From a PDT perspective, these results are significant because they show that hydrophobicity can be increased without preventing singlet oxygen generation. Together with the partition coefficient (Section 3.2.3) and solvatochromic analyses, the data support the view that a controlled modulation of hydrophobicity is beneficial when it enhances affinity for less polar membrane-like environments without promoting aggregation-dominated regimes. In addition, the modest bathochromic shifts observed after esterification are favorable for excitation at longer wavelengths, potentially enabling deeper light penetration into biological tissues. The preservation of singlet oxygen generation in most organic media, combined with the increased hydrophobicity of the derivatives, suggests compatibility with membrane-like and nanostructured environments, whereas the aqueous results emphasize the importance of controlling aggregation. Collectively, these characteristics highlight the potential of RBOCT and RBDOD as promising candidates for PDT applications.^10,25^

#### 3.2.2 Aggregation Behavior in Water/Ethanol Mixtures

The aggregation behavior of the ester derivatives RBOCT and RBDOD was investigated in water/ethanol mixtures with increasing water content (0–100%, v/v). Electronic absorption and fluorescence emission spectra were recorded at two time points (5 min and 24 h) to evaluate the initial aggregation process and its possible evolution over time. Figure 4 presents the absorption and emission spectra of RBOCT and RBDOD recorded at different water fractions after 24 h of equilibration.

**Figure 4:**
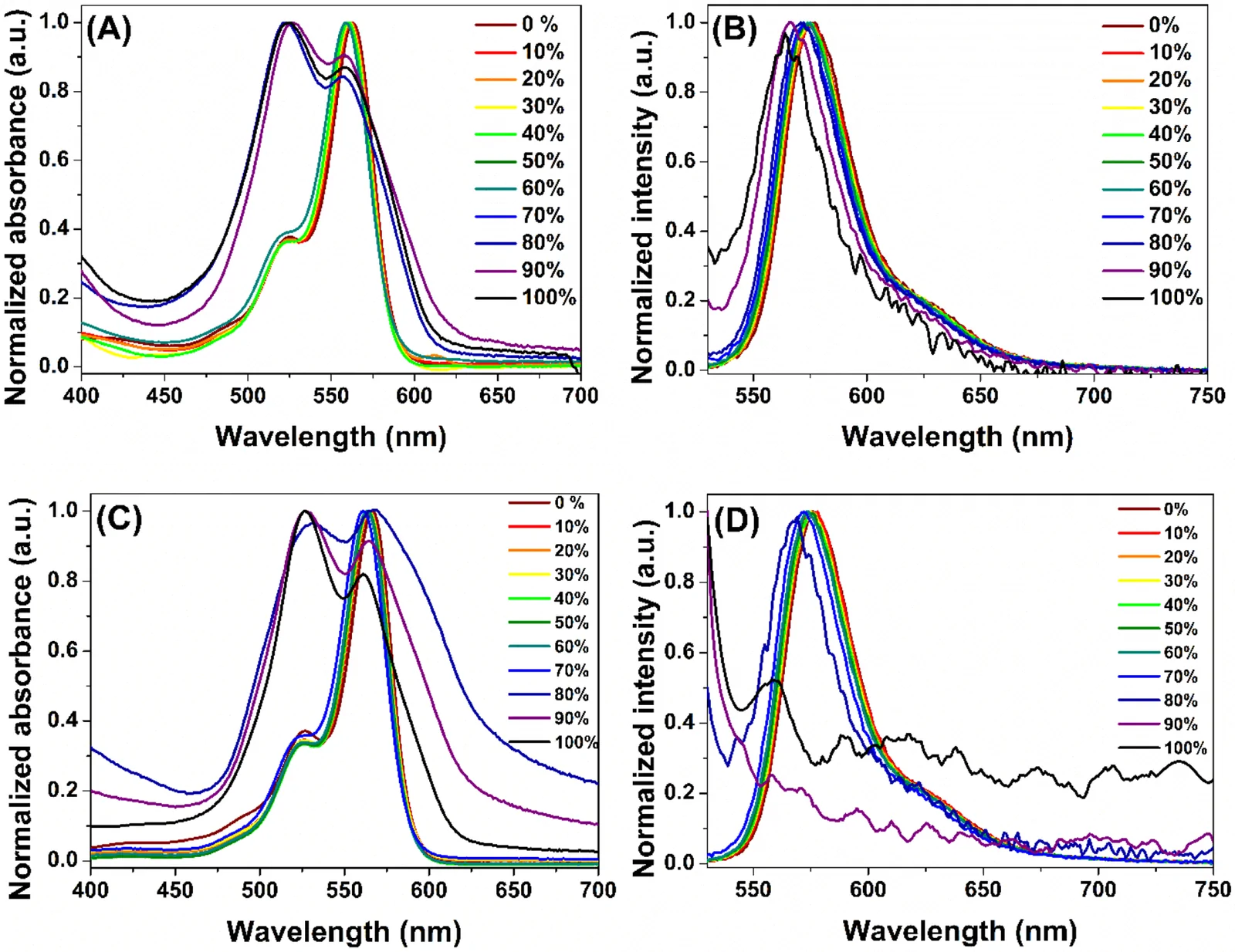
Electronic absorption spectra of RBOCT (A) and RBDOD (C), and fluorescence emission spectra of RBOCT (B) and RBDOD (D) in water/ethanol mixtures with different water volume fractions (% v/v), measured after 24 h. In water-rich media, RBDOD emission is very weak and appears as a noisy baseline due to extensive aggregation and quenching.

In water-rich media, the absorption bands of both compounds are markedly broadened and exhibit pronounced hypochromism, accompanied by a significant decrease in the absorbance intensity and increased baseline scattering. These spectral features are characteristic of self-aggregation phenomena and have been widely reported for ester derivatives of xanthene dyes and related hydrophobic PSs under aqueous conditions.^9,10,19^ In contrast, in ethanol-rich mixtures, the absorption bands are narrower and well-defined, indicative of predominantly monomeric species and improved solubilization of the ester derivatives.

The corresponding fluorescence emission spectra (Figure 4B,D) reveal a strong suppression of emission intensity in water-rich media for both compounds. This behavior arises from a combination of factors, including a reduction in effective molar absorptivity, the formation of nonemissive aggregates, and enhanced nonradiative deactivation pathways promoted by aggregation.^10,57^ In ethanol-rich mixtures, the fluorescence intensity increases significantly, consistent with the predominance of monomeric species. Similar behavior has been described for other xanthene ester derivatives in ethanol-rich environments, in which dissociation in more ethanolic solutions restores emission.^19^

To quantitatively evaluate the onset of aggregation, the evolution of fluorescence emission as a function of water content was analyzed (Figure 5), allowing estimation of the critical water aggregation percentage (CWAP), commonly defined as the water fraction above which aggregated species become predominant.^28^ Below the CWAP, the spectra closely resemble those recorded in pure ethanol, with only minor variations in band position and intensity. Above this limit, the spectroscopic changes described above become markedly more pronounced, indicating the rapid formation of higher-order aggregates. The CWAP values were estimated to be approximately 80% for RBOCT and 70% for RBDOD, reflecting the enhanced hydrophobic effect promoted by longer dodecyl chains. This trend is fully consistent with the partition coefficient measurements discussed below, which demonstrate the greater lipophilic character of RBDOD relative to RBOCT.^10,15^ This behavior indicates that, in water-rich environments, longer alkyl chains favor compact chromophore packing into H-type or mixed aggregates, leading to reduced effective molar absorptivity and severe quenching of fluorescence emission.^19^ These findings are also consistent with the reduced singlet oxygen generation observed for the derivatives in aqueous media (Table 3).

**Figure 5:**
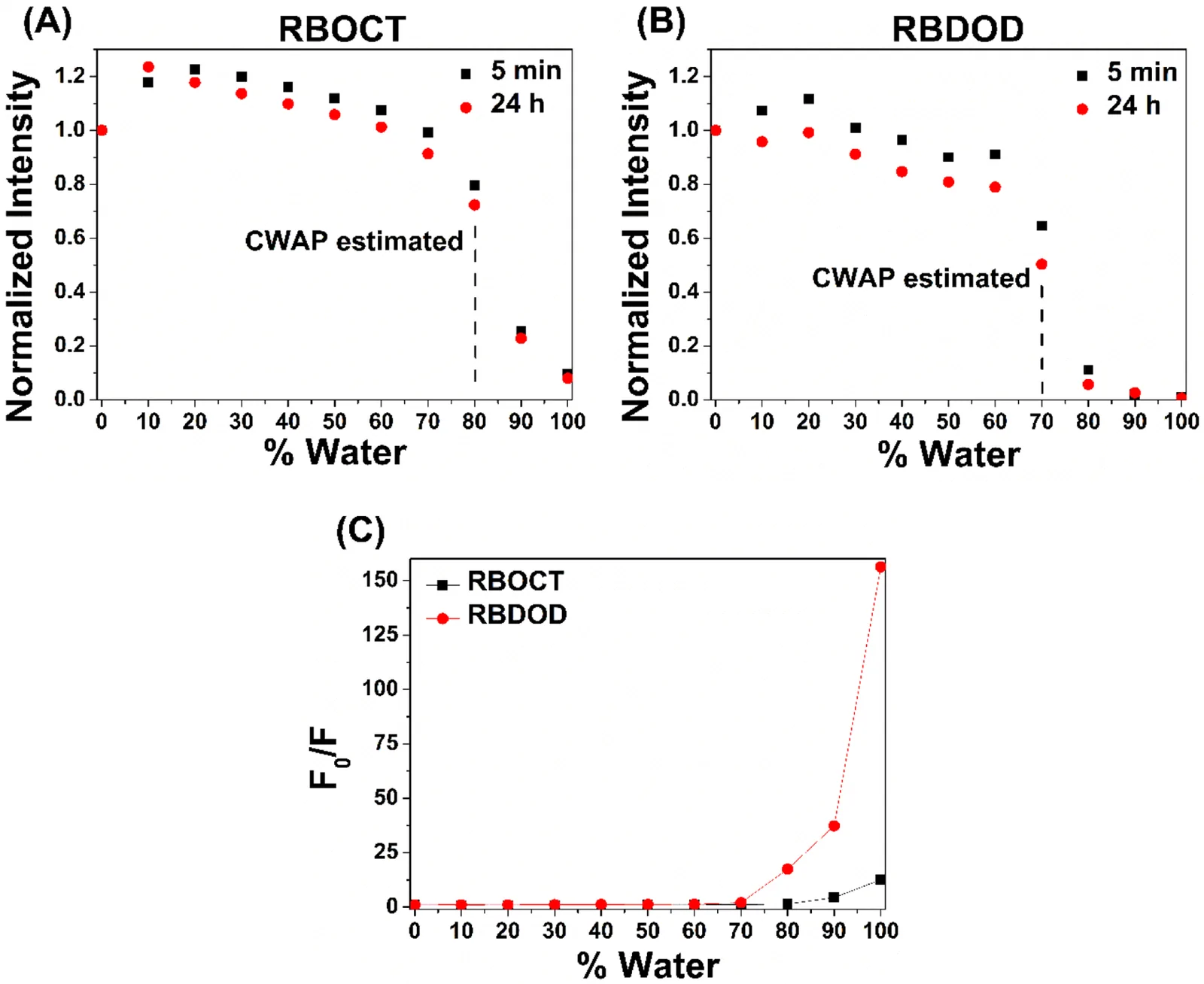
Dependence of normalized fluorescence emission intensity (*I*_ethanol_/*I*) for (A) RBOCT and (B) RBDOD in water/ethanol mixtures after 5 min and 24 h. Vertical dashed lines indicate the estimated CWAP. (C) Comparison of emission quenching expressed as *F*_0_/*F* (*F*_0_ = emission intensity in ethanol) for RBOCT and RBDOD after 24 h, highlighting the stronger aggregation propensity of the dodecyl-substituted derivative.

The aggregation behavior was further evaluated by comparing fluorescence emission spectra recorded after 5 min and 24 h of equilibration (Figure 5A,B). The overall spectral profiles are very similar at both times, indicating that aggregation occurs rapidly after solvent mixing, with only minor spectral evolution observed after 24 h. This behavior suggests that a quasi-equilibrium distribution of aggregated species is established within the first few minutes. Minor changes observed at longer times suggest slow structural rearrangements within the aggregates rather than the formation of new aggregate species.

Overall, RBDOD exhibits a greater tendency toward self-association than does RBOCT, consistent with its longer alkyl chain and higher hydrophobicity. Although aggregation leads to fluorescence loss and reduced apparent absorptivity in aqueous media, these results highlight the importance of microheterogeneous environments, such as micelles, lipid membranes, or polymeric carriers, for preserving the monomeric and photoactive forms of these ester derivatives in biological or aqueous media. This behavior is fully consistent with previous reports on hydrophobic xanthene-based PSs and reinforces the relevance of encapsulation strategies to recover the emissive monomeric state in water, thereby improving photodynamic efficiency.^10,15,58^

#### 3.2.3 Partition Coefficient and Hydrophobicity Assessment

The distribution of RB and its ester derivatives between 1-octanol and water was quantified by the partition coefficient (*K*_p_), expressed as log *K*_p_, which provides a quantitative description of lipophilicity and the effective hydrophobic–hydrophilic balance of the PSs under the experimental conditions.^59^ The experimental *K*_p_ and log *K*_p_ values are summarized in Table 4.

**Table 4:** Octanol/Water Partition Coefficients (*K*_p_) and log *K*_p_ Values of RB, RBOCT, and RBDOD^a^

	RB	RBOCT	RBDOD
*K*_P_	4.1 ± 0.1	46 ± 8	194 ± 47
log *K*_p_	0.62 ± 0.01	1.65 ± 0.07	2.27 ± 0.10

a Values are reported as mean ± standard deviation (*n* = 3).

At physiological pH (7.2–7.4), RB exists predominantly as a dianionic species, whereas esterification of the carboxylate group affords less charged derivatives, leading to a substantial increase in hydrophobicity. This reduction in net charge, combined with the introduction of hydrophobic alkyl chains, significantly alters the partitioning behavior of the dyes. According to operational criteria commonly used in PDT, PSs with log *K_p_* values in the range 0–1.5 can be considered amphiphilic, whereas values above 1.5 indicate predominantly hydrophobic character.^18,60^ In this context, RB exhibits lower affinity for the organic phase, whereas the two ester derivatives show increased partitioning into 1-octanol.

As expected, the partition coefficients increase consistently with the length of the alkyl chain, following the order RB < RBOCT < RBDOD (Table 4). This trend reflects the increasing strength of the hydrophobic effect associated with the octyl and dodecyl chains and is consistent with observations reported for alkyl ester derivatives of other xanthene dyes, such as eosin and erythrosine.^10,13,18^ In these systems, extension of the alkyl chain and the concomitant reduction in ionic character similarly promote a greater affinity for nonpolar environments.

In addition to the chain length, the high degree of halogenation in the xanthene framework also contributes to increased hydrophobicity. It has been reported that RB derivatives exhibit greater hydrophobicity than the less-halogenated eosin and erythrosine analogues under comparable conditions. This behavior has been attributed to the higher degree of halogen substitution in RB, which increases molecular polarizability and favors solvation in less polar media.^11^ These structural characteristics collectively reinforce the lipophilic nature of the RB ester derivatives.

The increase in hydrophobicity inferred from the log *K_p_* values closely correlates with the photophysical behavior discussed in the previous sections. The partitioning behavior is fully consistent with the aggregation trends observed in the water/ethanol mixtures. From a PDT standpoint, this amphiphilic-hydrophobic balance is particularly desirable, as it promotes interaction with biological membranes while enabling incorporation into delivery systems such as micelles or polymeric nanoparticles.^10,61^ Thus, the partitioning data not only corroborate the photophysical trends discussed above but also support the potential of RBOCT and RBDOD as promising RB-based photosensitizers for PDT applications.

### 3.3 Characterization in Biomimetic Micellar Systems

#### 3.3.1 Physicochemical Characterization of Micellar Systems

The incorporation of PSs into micelles and other nanocarrier systems plays an important role in the development of optimized formulations for PDT, as these assemblies enhance the solubilization, stabilization, and photoprotection of hydrophobic PSs under physiological conditions.^62,63^ This strategy has been widely reported for several classes of PSs, including porphyrins, phthalocyanines, and xanthene dyes, where micellar incorporation significantly improves solubility and photostability in aqueous media.^63,64^

The interaction of RB, RBOCT, and RBDOD with biomimetic micellar systems was investigated using a variety of surfactants of different charges and interfacial properties, including anionic SDS, cationic CTAB, nonionic TX-100, and zwitterionic SB3-14 (Table 1). These surfactants provide microenvironments with different polarities, charge densities, and hydration profiles, enabling a systematic evaluation of how such parameters influence PS aggregation and photophysical behavior in microheterogeneous media.^10,62^

To establish the physicochemical basis for interpreting the behavior of the PSs in biomimetic microheterogeneous media, the micellar systems were systematically characterized. DLS measurements revealed that all formulations exhibit *D*_h_ values in the nanometer range (6.3–17.2 nm; Table 5), characteristic of surfactant-based micelles.^65^ Blank micelles showed *D*_h_ values following the order CTAB < SDS < SB3–14 < TX-100, reflecting differences in surfactant molecular architecture, chain packing efficiency, and headgroup hydration.^66^ After incorporation of RB and its alkyl derivatives, only minor variations in *D*_h_ were observed, indicating that association of the PSs does not induce significant micellar growth or secondary aggregation.^12,56^ In the case of SDS/RBOCT, a slight decrease in *D*_h_ was observed, which likely reflects changes in the hydration layer rather than structural rearrangement of the micelles.^12,67^

**Table 5:** Hydrodynamic Diameter (*D*_h_), PDI, ζ Potential, and Micellar Encapsulation Efficiency (EE) of Blank and PS-Loaded Micellar Systems

Surfactant	PS	*D*_h_ (nm)	PDI	ζ (mV)	EE (%)
**SDS**	Blank	7.9 ± 0.8	0.363 ± 0.025	-78.4 ± 9.7	-
	RB	12.4 ± 0.1	0.393 ± 0.033	-99.9 ± 4.7	94.4
	RBOCT	6.7 ± 1.0	0.460 ± 0.054	-93.6 ± 7.4	99.9
	RBDOD	10.2 ± 0.9	0.329 ± 0.141	-87.9 ± 5.8	99.9
**CTAB**	Blank	6.3 ± 0.1	0.210 ± 0.017	+24.1 ± 1.0	-
	RB	10.0 ± 0.2	0.385 ± 0.012	+13.2 ± 2.0	99.9
	RBOCT	9.1 ± 0.5	0.131 ± 0.008	+13.7 ± 9.6	99.7
	RBDOD	8.5 ± 0.3	0.221 ± 0.005	+33.8 ± 4.0	99.9
**TX-100**	Blank	11.4 ± 0.2	0.205 ± 0.008	-3.1 ± 1.9	-
	RB	17.2 ± 0.9	0.275 ± 0.018	-7.3 ± 3.0	99.3
	RBOCT	16.8 ± 0.4	0.263 ± 0.018	-3.5 ± 2.8	98.8
	RBDOD	13.5 ± 1.3	0.244 ± 0.006	-4.6 ± 1.3	99.8
**SB3–14**	Blank	10.9 ± 0.4	0.327 ± 0.072	-11.1 ± 0.7	-
	RB	10.7 ± 0.7	0.280 ± 0.019	-12.2 ± 3.3	99.2
	RBOCT	11.7 ± 1.1	0.258 ± 0.061	-16.8 ± 4.5	99.9
	RBDOD	10.3 ± 1.5	0.221 ± 0.007	-15.6 ± 1.8	99.4

The micellar homogeneity was further evaluated by the polydispersity index. PDI values ranged from 0.21 to 0.46 (Table 5), consistent with the intrinsic polydispersity of surfactant-based micellar systems.^66^ CTAB, TX-100, and SB3-14 formulations exhibited comparatively lower PDI values, indicating greater population uniformity, whereas SDS systems consistently exhibited higher dispersion, especially for the SDS/RBOCT system. This behavior aligns with previous reports describing a broader size distribution for anionic micelles relative to cationic, nonionic, and zwitterionic systems.^66^

Zeta potential measurements provided further insight into the interfacial organization of the micellar assemblies. SDS-based micelles exhibited highly negative ζ potentials (−78.4 to −99.9 mV), consistent with the strong anionic character of dodecyl sulfate headgroups.^68^ Incorporation of RB further increased the magnitude of the negative ζ potential, indicating a measurable contribution of the dianionic PS to the electrostatic properties of the micellar interface. In contrast, the RBOCT and RBDOD yielded slightly less negative ζ potentials, suggesting reduced exposure of the chromophore charge to the aqueous phase.^10,12,67^

CTAB micelles exhibited positive ζ potentials, as expected for quaternary ammonium headgroups.^69^ While the blank system exhibited a ζ potential of +24.1 mV, incorporation of RB and RBOCT reduced this value to approximately +13 mV, consistent with the contribution of the negatively charged chromophore to the interfacial electrostatic environment.^67^ In contrast, the CTAB/RBDOD system exhibited an increased ζ potential (+33.8 mV), which may indicate a reduced contribution of the chromophore charge to the micellar surface, possibly due to deeper insertion of the more hydrophobic derivative within the assembly.^10,67^

TX-100 and SB3-14 micelles exhibited ζ potentials close to neutrality. TX-100 systems exhibited values between −3.1 and −7.3 mV, consistent with the nonionic nature of poly­(oxyethylene)-based surfactants, whereas SB3-14 micelles exhibited slightly negative ζ potentials characteristic of sulfobetaine zwitterionic interfaces.^70^ From a colloidal standpoint, the high absolute ζ potentials observed for SDS and CTAB formulations indicate predominantly electrostatic stabilization, whereas TX-100 and SB3-14 micelles are mainly stabilized by steric and hydration effects associated with their bulky hydrophilic headgroups.^71^

EE values were consistently high (>94%) across all micellar systems, with most formulations exhibiting EE values above 99% (Table 5), demonstrating strong partitioning of RB and its alkyl ester derivatives into the surfactant assemblies.^10,67^ In SDS micelles, EE increased with PS hydrophobicity (from 94.4% for RB to 99.9% for RBDOD).^67^ In contrast, CTAB, TX-100, and SB3-14 micelles exhibited uniformly high EE values (>98%), regardless of PS hydrophobicity, consistent with efficient hydrophobic partitioning into less hydrated micellar regions.^18,19^

Overall, these physicochemical results indicate that CTAB, TX-100, and SB3-14 provide biomimetic microenvironments with surface charges and hydrophobicities suitable for lipophilic PS insertion and photophysical recovery. In contrast, SDS represents a model system dominated by electrostatic repulsion, mimicking interfacial conditions where penetration of amphiphilic PSs is restricted.^26^

#### 3.3.2 Spectral Profiles and Determination of Apparent p*K*_a_ in Micellar Media

Before evaluating the photophysical properties of RB and its derivatives in biomimetic micellar systems, it is essential to establish which protolytic and tautomeric species predominate under physiological conditions (pH 7.4). In general, the determination of p*K*_a_ values for halogenated xanthene dyes is challenging due to the extensive spectral overlap among protolytic species (Figure 6) and the proximity of their p*K*_a_ values.^10,43^ In addition, these compounds involve complex protolytic and tautomeric equilibria (Scheme S1, Supporting Information). For RB, four main protolytic species have been described depending on pH and medium polarity: cationic, neutral, monoanionic, and dianionic forms.^72,73^

**Figure 6:**
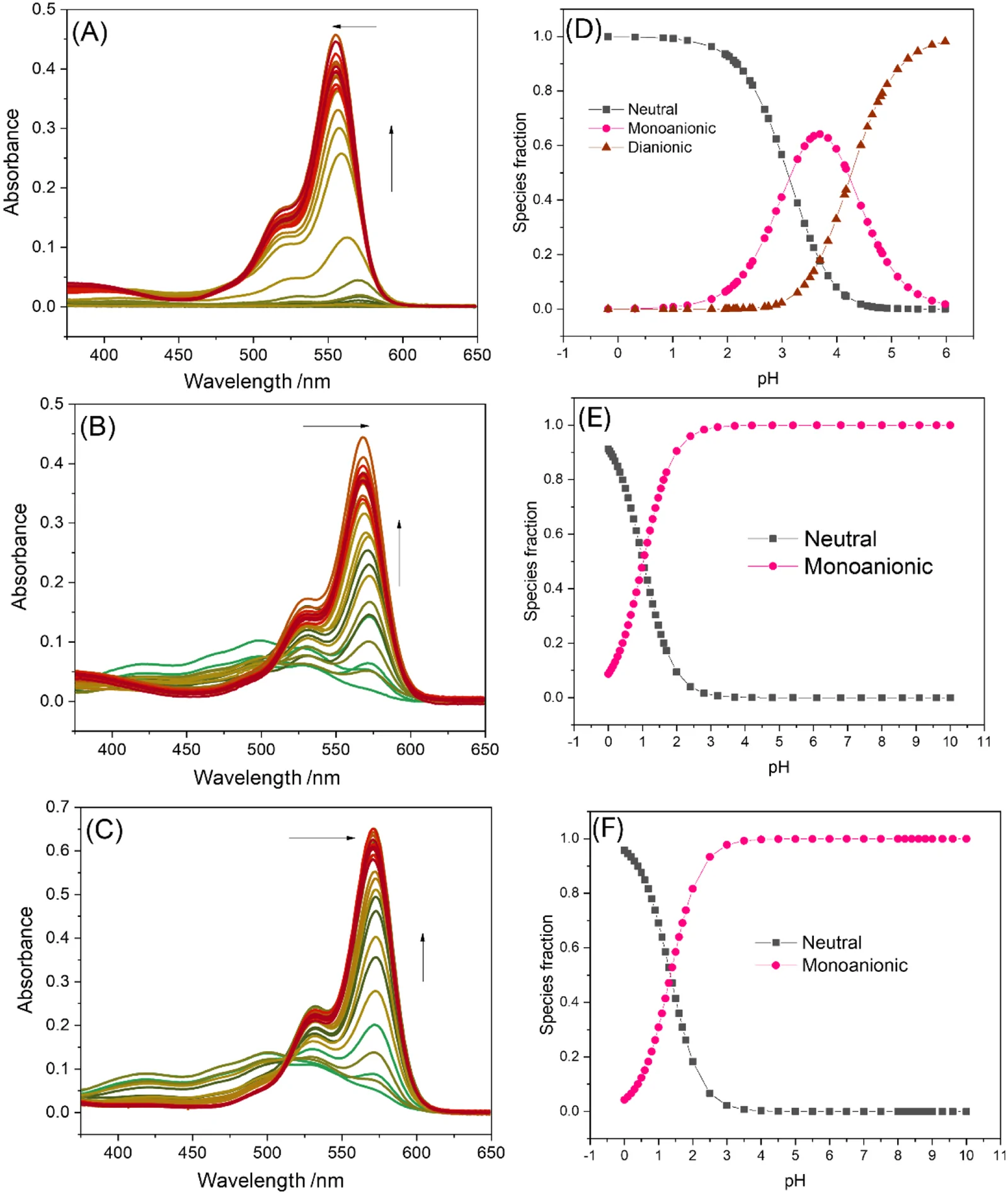
Absorption spectra in TX-100 micellar medium at various pH (A) RB, (B) RBOCT, (C) RBDOD, and relative concentration of each protolytic species at various pH for (D) RB, (E) RBOCT, and (F) RBDOD; [PS] = 5.0 × 10^–6^ mol L^–1^, TX-100 = 1.0% (w/v), and 25.0 °C. The arrows indicate the spectral changes with increasing pH.

The neutral form may exist as different tautomers, mainly the lactone (NEL) and quinoid (NEQ) structures, as previously described for RB and related xanthene dyes.^74^ In polar media, such as aqueous solutions, NEQ is favored, whereas NEL predominates in less-polar and aprotic environments. NEL formation disrupts the conjugation of the xanthene ring due to sp^3^ hybridization of the central carbon atom, leading to loss of the characteristic chromophore coloration.^75,76^

For the monoanionic species, two main tautomers have been proposed: the carboxylate monoanion (MAC) and the phenolate monoanion (MAF). In water, the MAC form predominates because the carboxylic group is more acidic than the phenolic group, favoring initial deprotonation at the carboxylate site.^72,75^ This behavior is particularly relevant for interpreting the acid–base equilibria of RB and its derivatives in different micellar microenvironments.

The complexity of the systems limits the applicability of univariate approaches based on isolated wavelengths or spectral derivatives.^43^ To overcome these limitations, p*K*_a_ values were determined by multivariate chemometric analysis using MASDA,^42,77^ as previously reported for xanthenes.^10,14,19^ In this approach, spectra collected over the entire pH range are simultaneously analyzed by principal component analysis (PCA), enabling the mathematical resolution of overlapping protolytic species. The molar fractions obtained for each species are then fitted to the Henderson–Hasselbalch equation to determine the p*K*_a_ values.

As a reference for the micellar systems, the p*K*_a_ values of RB in water obtained by MASDA were adopted.^72^ The analysis explained 99.908% of the spectral variance, revealing three protolytic species with p*K*_a_ values of 1.89 and 3.93. Based on the more pronounced UV–Vis spectral changes associated with protonation of the xanthene phenolate group relative to the benzene carboxylate group, the p*K*_a_ values were assigned to the carboxylic (1.89) and phenolic (3.93) groups, respectively. Equilibria at pH < 0 were not investigated.

For RB, all evaluated micellar systems exhibited very low or nearly absent electronic absorption under acidic conditions, accompanied by the disappearance or strong reduction of characteristic dye coloration. This behavior indicates the predominance of NEL species, which is favored in low-polarity environments such as the micellar core. These results agree with the high incorporation efficiencies obtained for RB in all surfactants, indicating a strong association between the PS and the hydrophobic micellar microenvironment.

As the pH increased, the characteristic pink coloration of RB gradually appeared, accompanied by a progressive increase in absorption intensity until stabilization at higher pH values, indicating a shift of the equilibrium toward the conjugated anionic species. As a representative example, Figure 6A shows the spectra of RB in TX-100. Notably, no isosbestic point was observed in any system, confirming the existence of complex equilibria.

For the RBOCT and RBDOD derivatives, the protolytic equilibrium was simpler due to the elimination of the equilibrium associated with carboxylic deprotonation and prevented the formation of the NEL species (Figure 6B,C). Unlike RB, both derivatives exhibited electronic absorption even under acidic conditions, accompanied by a faint pink coloration and absorption bands shifted to shorter wavelengths, indicating the predominance of the NEQ species. In this case, esterification prevents lactone ring closure, preserving partial conjugation of the xanthene system, even at low pH.

With increasing pH, a bathochromic shift of the absorption bands was observed (Δλ ≈ 65 nm), accompanied by a significant increase in spectral intensity. This behavior indicates progressive formation of deprotonated phenolic species, which are more electronically conjugated and therefore promote greater electron delocalization within the xanthene chromophore. Furthermore, the spectral changes observed for the derivatives were less abrupt than those of RB, reflecting the simplification of the protolytic system after esterification of the carboxylic group.

From the MASDA output files, the relative concentrations of the species involved in each protolytic system were calculated to determine the apparent p*K*_a_ values (Figure 6D-F). Owing to alkylation of the benzene ring, the protolytic equilibria of the ester derivatives allow direct evaluation of the neutral–monoanionic equilibrium (p*K*_a1_) within the conventional pH range. It is important to note that the experimental p*K*_a_ values obtained in this study are influenced by the colloidal environment. Therefore, the reported values are referred to as apparent p*K*_a_ values (Table 6).

**Table 6:** Apparent p*K*_a_ Values of RB and Its Derivatives in Micellar Media

PS		TX-100	CTAB	SDS	SB3-14
**RB**	p*K*_aCOOH_	4.2	1.4^a^	4.4^a^	2.9
	p*K*_aOH_	3.1	0.1^a^	2.6^a^	1.5
**RBOCT**	p*K*_aOH_	1.0	0.8	2.4	1.1
**RBDOD**	p*K*_aOH_	1.4	0.9	2.5	0.8

a Literature values were obtained from Pereira.^10^

For RB, the apparent p*K*_a_ values across all micellar systems revealed an inversion in the acidity order of the protolytic groups, with p*K*_aOH_ < p*K*_aCOOH_. This behavior differs from that typically observed in aqueous media, where the carboxylic group is more acidic than the phenolic group.^72^ The inversion indicates a strong influence of the micellar microenvironment on the protolytic and tautomeric equilibria of RB, highlighting the dye’s interactions with different colloidal structures.

This effect can be rationalized by the lower polarity of the micellar cores. In low-polarity environments, the carboxylic group, particularly in its ionized form, becomes less efficiently solvated, making deprotonation less favorable and increasing the p*K*_aCOOH_. In contrast, the phenolate charge generated after OH deprotonation is extensively delocalized by resonance throughout the xanthene system, stabilizing the deprotonated species even in less-polar media and preserving the relatively high acidity of the phenolic group. Consequently, the relative decrease in carboxylic acidity leads to the observed inversion in ionization order.^10,14,19^

In the nonionic surfactant TX-100, this inversion was clearly observed with p*K*_aCOOH_ = 4.2 and p*K*_aOH_ = 3.1. In the absence of surface charges, the observed effects mainly arise from the low polarity of the micellar core and the ethoxylated interfacial region. These results suggest that RB is preferentially located in a micellar region (Section 3.4.1) of intermediate polarity, sufficiently hydrophobic to reduce carboxylate solvation and promote p*K*_a_ inversion, but without the strong electrostatic effects present in ionic surfactants.

In the cationic surfactant CTAB, the lowest apparent p*K*_a_ values were observed, particularly for p*K*_aOH_ (∼0.1).^10^ In this system, the positively charged micellar surface strongly stabilizes the anionic species formed after deprotonation, favoring RB ionization. In addition, the hydrophobic and less hydrated environment of CTAB micelles further reduces carboxylic group solvation, enhancing the acidity inversion effect. This behavior agrees with previous studies of xanthene dyes in cationic micelles, where electrostatic interactions combined with low microenvironment polarity were identified as key factors governing p*K*_a_ inversion.^10,78^

In contrast, the anionic surfactant SDS exhibited the highest p*K*_aOH_ (2.6) and p*K*_aCOOH_ (4.4) values. The negatively charged micellar surface disfavors the formation of the RB anionic species through electrostatic repulsion, shifting the deprotonation equilibria toward higher pH values. Furthermore, the more hydrated SDS interface partially stabilizes ionized forms, resulting in p*K*_a_ values closer to those observed in an aqueous solution, although the acidity inversion remained evident.

The zwitterionic surfactant SB3-14 showed intermediate behavior between CTAB and SDS, with p*K*_aCOOH_ = 2.9 and p*K*_aOH_ = 1.5. The coexistence of positive and negative charges in the polar headgroup creates an interface with a lower effective charge density, minimizing extreme electrostatic effects. As a result, the RB protolytic equilibria are governed mainly by the local polarity and micellar interfacial organization. Consequently, the acidity inversion is maintained, although with p*K*_a_ shifts smaller than those observed in CTAB.

The esterified derivatives RBOCT and RBDOD exhibited only one protolytic equilibrium associated with the phenolic group because of esterification of the carboxylic moiety. In all surfactants, the p*K*_aOH_ values remained relatively low, indicating that the increased hydrophobicity introduced by the alkyl chains promotes deeper insertion of the derivatives into the micellar core. This effect preferentially exposes the phenolic group to less-polar environments, stabilizing the deprotonated species and favoring ionization. Moreover, the small differences between RBOCT and RBDOD suggest that increasing the alkyl chain length slightly enhances hydrophobic association with the micelles, particularly in cationic and zwitterionic systems.

Finally, considering the apparent p*K*_a_ values obtained in the micellar systems, all compounds are expected to be predominantly in their anionic forms at a physiological pH (7.4). Under these conditions, the deprotonated species are strongly favored, resulting in fully conjugated xanthene structures with intense visible absorption, which is particularly relevant to their photophysical and photodynamic properties. In contrast, under mildly acidic environments, such as those found in inflamed tissues, intracellular vesicles, or the tumor microenvironment, partial protonation of the dyes may occur, shifting the equilibria toward neutral or monoanionic species. These changes can significantly affect the electronic spectra, aggregation behavior, membrane interactions, and ROS generation efficiency of xanthene PSs, as previously reported for related systems.^79^

#### 3.3.3 Apparent Spectra of Protolytic Species and Their Molar Absorptivities

One particularly valuable feature of the chemometric approach is its ability to reconstruct the apparent spectra of the species involved in the equilibria, as illustrated in Figure 7 for RB and its derivatives in micellar systems. Table 7 summarizes the corresponding λ_max_ and apparent molar absorptivity (ε) values for each protolytic species.

**Figure 7:**
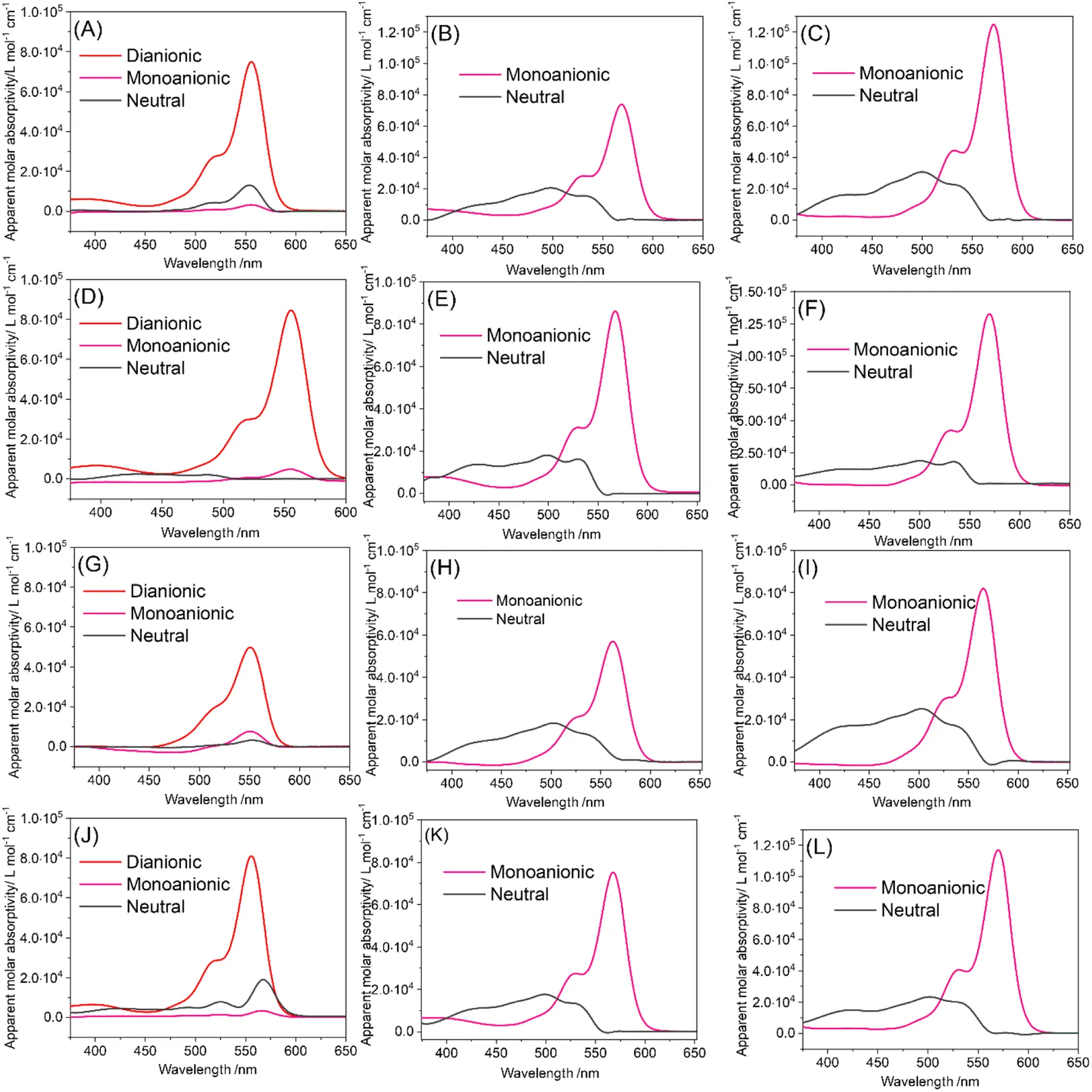
Apparent spectra of the species calculated by the K-matrix method: (A) RB in TX-100, (B) RBOCT in TX-100, (C) RBDOD in TX-100, (D) RB in CTAB, (E) RBOCT in CTAB, (F) RBDOD in CTAB, (G) RB in SDS, (H) RBOCT in SDS, (I) RBDOD in SDS, (J) RB in SB3–14, (K) RBOCT in SB3–14, and (L) RBDOD in SB3–14. [PS] 5.0 × 10^–6^ mol L^–1^, [surfactant] = 1.0% (w/v) and 25 °C.

**Table 7:** Wavelength and Molar Absorptivity Values for the Pure Protolytic Species Obtained Using the K-Matrix Method

Surfactant	PS				Specie	λ_max_ (nm)	ε (10^3^ L mol^–1^cm^–1^)
**TX-100**	RB				Neutral	556.0	3.7
					Monoanionic	554.3	13.4
					Dianionic	555.7	91.8
	RBOCT				Neutral	498.1	20.6
					Monoanionic	568.5	88.8
	RBDOD				Neutral	500.2	31.0
					Monoanionic	571.1	125.2
**CTAB**	RB	Neutral				488.4	2.1
		Monoanionic				554.9	4.8
		Dianionic				555.1	93.4
	RBOCT	Neutral				497.9	18.4
		Monoanionic				567.4	102.0
	RBDOD	Neutral				500.2	19.4
		Monoanionic				569.8	133.1
**SDS**	RB	Neutral				552.7	3.2
		Monoanionic				550.0	7.7
		Dianionic				551.1	80.0
	RBOCT	Neutral				502.7	18.6
		Monoanionic				561.8	80.0
	RBDOD	Neutral				503.0	25.2
		Monoanionic				564.7	99.8
**SB3–14**	RB		Neutral			565.8	4.0
			Monoanionic			568.2	19.0
			Dianionic			556.2	81.0
	RBOCT		Neutral			498.1	18.0
			Monoanionic			568.0	83.0
	RBDOD			Neutral		501.8	23.6
				Monoanionic		570.4	117.8

Overall, the spectral data demonstrate a strong influence of the micellar microenvironment on the protolytic and tautomeric equilibria of the dyes, affecting both the band position and absorption intensity.

For RB, the neutral species exhibited low molar absorptivity in all surfactants, with ε values ranging from 2.1 × 10^3^ to 4.0 × 10^3^ L mol^–1^ cm^–1^. This behavior is associated with the predominance of NEL species, whose structure partially disrupts the electronic conjugation of the xanthene system, markedly reducing absorption intensity. This effect was particularly pronounced in CTAB, where the neutral species showed the lowest ε value (2.1 × 10^3^ L mol^–1^ cm^–1^) together with a significant hypsochromic shift (λ_max_ = 488.4 nm). These results indicate strong stabilization of NEL in the cationic and poorly hydrated micellar environment, favored by the low polarity of the micellar core.

In contrast, in SDS, TX-100, and SB3-14, the neutral RB species displayed absorption bands at longer wavelengths (∼553–566 nm), suggesting lower disruption of the chromophoric system relative to CTAB. Nevertheless, the low ε values confirm that the NEL tautomer remained predominant. In SDS, the more hydrated interface and the anionic character of the micelle likely reduce deep insertion of RB into the hydrophobic core, leading to spectral features closer to those observed in aqueous solution. Intermediate effects observed for TX-100 and SB3-14 reflect fewer extreme microenvironments in terms of polarity and electrostatic interactions.

The dianionic RB species displayed the highest molar absorptivity values in all evaluated systems. In this form, the fully conjugated phenolate group promotes extensive electron delocalization and strong visible absorption. The λ_max_ values remained close to 555 nm regardless of the surfactant, indicating that the dianionic species is less sensitive to the micellar microenvironment than the neutral species.

The esterified derivatives RBOCT and RBDOD showed spectral behavior markedly different from RB. Esterification of the carboxylic group makes the formation of the NEL structure sterically unfavorable, preventing the existence of these tautomers. Consequently, the neutral species of these derivatives predominantly adopt a NEQ structure, preserving conjugation within the xanthene system. As a result, the neutral ester species exhibited higher molar absorptivity values than RB. In addition, the λ_max_ values of the neutral ester derivatives remained nearly constant across all surfactants, indicating that the micellar environment exerts a weaker effect on these species than on RB. This behavior suggests that elimination of the NEL/NEQ equilibrium significantly reduces spectral sensitivity to medium polarity.

The esterified derivatives RBOCT and RBDOD showed spectral behavior markedly different from RB. Esterification of the carboxylic group makes the formation of the NEL structure sterically unfavorable, preventing the existence of these tautomers. Consequently, the neutral species of these derivatives predominantly adopt an NEQ structure, preserving conjugation within the xanthene system. As a result, the neutral ester species exhibited higher molar absorptivity values than RB. In addition, the λ_max_ values of the neutral ester derivatives remain nearly constant across all surfactants, indicating that the micellar environment exerts a weaker effect on these species than on RB. This behavior suggests that elimination of the NEL/NEQ equilibrium significantly reduces spectral sensitivity to medium polarity.

The monoanionic species of RBOCT and RBDOD exhibited the highest molar absorptivity values of the entire data set, reaching up to 133.1 × 10^3^ L mol^–1^ cm^–1^ for RBDOD in CTAB. The strongly conjugated phenolate moiety substantially increases electron density within the π-system, resulting in intense electronic absorption and bathochromic shifts to ∼562–571 nm.

Overall, ε values for the monoanionic species increased in the order SDS < SB3–14 < TX-100 < CTAB. This trend highlights the role of micellar polarity and electrostatic interactions in stabilizing phenolate species. In CTAB, the cationic surface efficiently stabilizes the anionic dye species, favoring greater electron delocalization and stronger absorption bands. In contrast, electrostatic repulsion between the anionic SDS headgroups and the deprotonated species reduces phenolate stabilization, resulting in lower ε values. Finally, RBDOD systematically exhibited higher molar absorptivity values than RBOCT, particularly for the monoanionic species.

### 3.4 Photophysical Behavior in Microheterogeneous Media

#### 3.4.1 Steady-State Absorption and Emission in Micellar Systems

To investigate the photophysical properties of RB and its derivatives in biomimetic micellar systems, all experiments were carried out in a PBS buffer (pH 7.4) to reproduce physiological conditions. As established in Section 3.3.2, RB is predominantly present as the dianion, whereas both RBOCT and RBDOD exist mainly as the monoanion in all biomimetic micellar systems studied.^10,19^

Upon micellar incorporation, the absorption and emission spectra of the PSs become consistent with monomeric species, as evidenced by the narrowing of spectral bands and attenuation of aggregation-related distortions observed in aqueous solution (Section 3.2.1). This behavior is illustrated in the normalized spectra shown in Figure 8. The corresponding photophysical parameters, including absorption and emission maxima (*λ*_abs_ and *λ*_*em*_) and Φ_*F*_, are summarized in Table 8.

**Figure 8:**
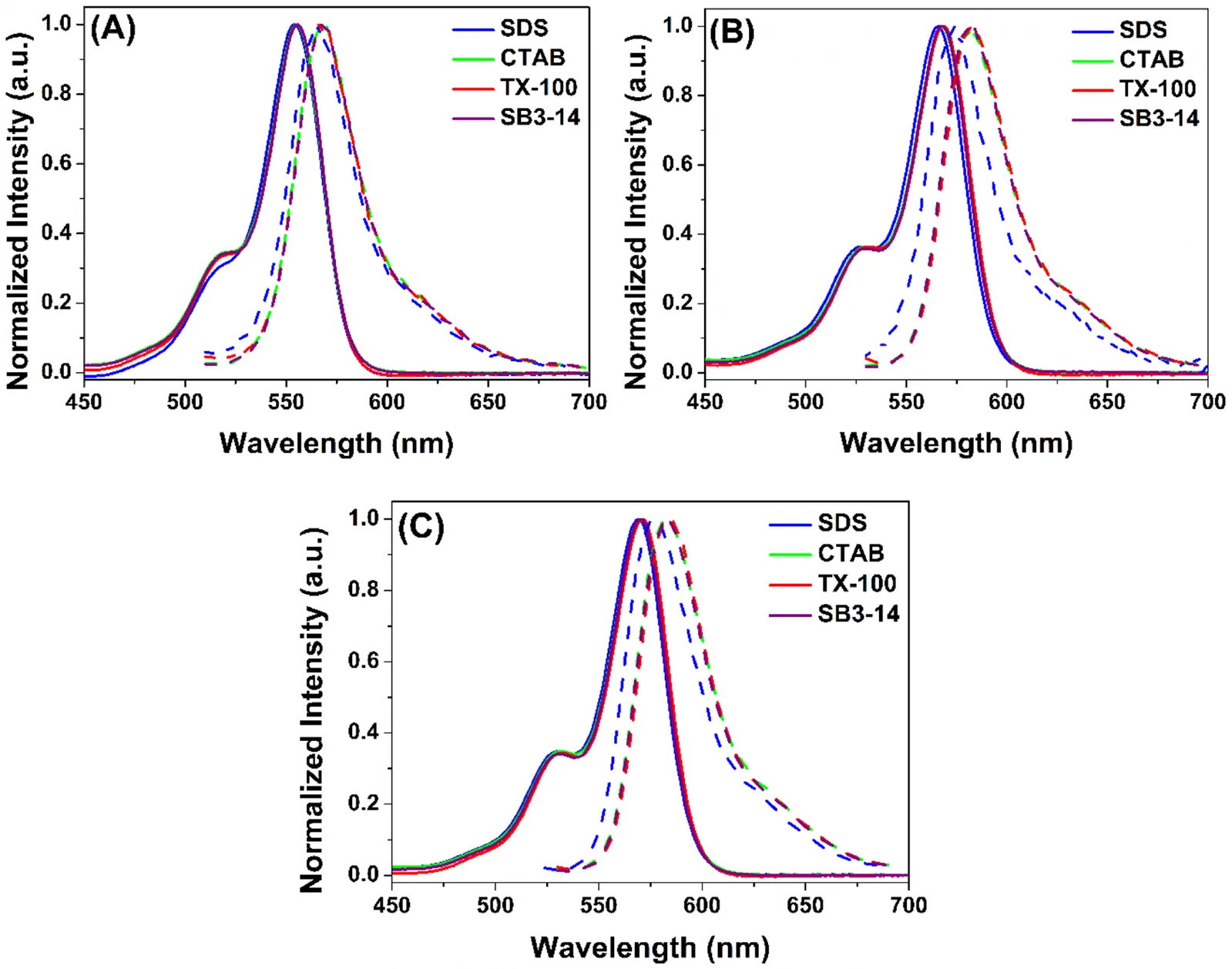
Normalized absorption (solid lines) and fluorescence emission (dashed lines) spectra of (A) RB, (B) RBOCT, and (C) RBDOD in SDS, CTAB, TX-100, and SB3–14 micelles (PBS, pH 7.4). Spectra are normalized to unity at *λ*_max_ to highlight changes in band position and spectral shape induced by the different micellar microenvironments.

**Table 8:** Steady-State Photophysical Parameters of RB, RBOCT, and RBDOD in Different Micellar Systems

Surfactant	PS	*λ*_abs_ (nm)	λ_em_(nm)	Φ_F_
**SDS**	RB	554	566	0.032 ± 0.005
	RBOCT	566	573	0.037 ± 0.002
	RBDOD	569	584	0.005 ± 0.001
**CTAB**	RB	555	567	0.052 ± 0.001
	RBOCT	568	582	0.113 ± 0.003
	RBDOD	570	584	0.148 ± 0.004
**TX-100**	RB	556	566	0.075 ± 0.002
	RBOCT	568	582	0.235 ± 0.002
	RBDOD	571	582	0.283 ± 0.003
**SB3-14**	RB	556	567	0.059 ± 0.002
	RBOCT	568	581	0.139 ± 0.002
	RBDOD	570	584	0.177 ± 0.003

In all micellar systems, the *λ*_abs_ values follow the same trend observed in homogeneous solvents (RB < RBOCT < RBDOD), indicating that the intrinsic electronic structure of the chromophores is preserved upon micellar incorporation. The spectral profiles obtained indicate efficient partitioning of the PSs into the assemblies under the experimental conditions, driven by a combination of hydrophobic and electrostatic effects that depend on the nature of the surfactant. SDS micelles exhibit the smallest bathochromic shifts among the micellar systems. Similar behavior has been reported for other xanthene derivatives, such as eosin and erythrosine, in SDS micelles.^13,67^

In contrast, CTAB, TX-100, and SB3-14 micelles induce slight red shifts, suggesting a greater extent of chromophore insertion into less hydrated and more apolar micellar domains.^10,18^ Comparable spectral behavior has been reported for eosin and fluorescein derivatives in nonionic and cationic micelles, where deeper chromophore insertion correlates with reduced interfacial hydration.^80,81^

Fluorescence emission measurements further corroborate effective PS partitioning and micelle-induced deaggregation.^10,67^ In aqueous solution, both RBOCT and RBDOD display strongly quenched emission due to aggregation and dominant nonradiative decay pathways associated with H-type aggregates.^82^ Upon micellar incorporation, fluorescence intensity increases markedly for most systems (Figure 8), reflecting recovery of emissive monomeric species. This behavior is quantitatively captured by the Φ_F_ values reported in Table 8, which increase sharply relative to water (Φ_F_ < 0.015), reaching their highest values in TX-100 micelles, followed by SB3-14 and CTAB. Among the micellar systems investigated, TX-100 consistently produced the highest fluorescence quantum yields for both ester derivatives, indicating more efficient recovery of the emissive monomeric species. This behavior is consistent with the nonionic character of TX-100 micelles, which appear to provide a microenvironment that minimizes aggregation and reduces nonradiative deactivation pathways.^67^

An exception is observed for RBDOD in SDS micelles, where fluorescence remains strongly suppressed (Φ_F_ = 0.005). This behavior may arise from the combined effects of electrostatic repulsion and the high hydrophobicity of the dodecyl-substituted derivative, which can favor residual aggregation or greater exposure of the chromophore to the aqueous environment.^67,80^

These results demonstrate that micellar systems are able to modulate the local environment experienced by the PSs and restore their monomeric photophysical behavior. However, fluorescence measurements alone do not provide direct information regarding the localization of the PSs within micellar assemblies. Since the spatial distribution of the PSs may strongly influence their interaction with molecular oxygen, Stern–Volmer experiments using iodide ions as water-soluble quenchers were performed to investigate the accessibility of the PSs within the different micellar microenvironments. The corresponding *K*_SV_ values obtained for the PSs and DPBF in the different micellar systems are presented in Table 9.

**Table 9:** Apparent Stern–Volmer Quenching Constants (*K*_SV_) Obtained for RB, RBOCT, RBDOD, and DPBF in SDS, CTAB, TX-100, and SB3-14 Micelles^a^

	*K*_SV_ (L mol^–1^)			
PS	TX-100	CTAB	SDS	SB3-14
**RB**	0.97	376.37	5.57	1.37
**RBOCT**	0.96	608.77	0.88	2.99
**RBDOD**	0.94	847.54	1.30	2.67

a All Stern–Volmer plots exhibited excellent linearity with *R* ^2^ values ranging from 0.991 to 0.999.

Iodide is commonly used as a water-soluble quencher to probe the fluorophore accessibility in organized media. However, in charged micellar systems, its local concentration near the micellar interface may differ from the analytical bulk concentration because of electrostatic and counterion effects. Therefore, the experimentally determined *K*_SV_ values provide an apparent measure of fluorophore accessibility within each micellar environment rather than an intrinsic interfacial quenching constant. In general, higher apparent *K*_SV_ values are consistent with greater quencher accessibility, whereas lower values indicate more effective shielding from the aqueous/interfacial environment.^83^

For Triton X-100 micelles, all PSs exhibited very low and nearly identical *K*_SV_ values (Table 9). These results indicate that all compounds are effectively shielded from the aqueous quencher and occupy similar protected microenvironments within the micellar structure. Despite the substantial increase in the hydrophobicity observed for the ester derivatives, no significant changes in accessibility were detected. The extensive polyoxyethylene layer characteristic of Triton X-100 likely contributes to the efficient shielding of the incorporated chromophores from the aqueous phase.

A markedly different behavior was observed in CTAB micelles, where the apparent *K*_SV_ values increased substantially in the order RB < RBOCT < RBDOD, paralleling the increase in hydrophobicity from RB to RBDOD. As discussed in Section 3.3.2, at pH 7.4 RB exists predominantly as a dianionic species, whereas RBOCT and RBDOD are mainly present as monoanionic species. Although these differences in molecular hydrophobicity and charge may influence the distribution and accessibility of the PSs within the micellar assemblies, the *K*_SV_ values in CTAB must also be interpreted considering the cationic nature of the micellar interface. Previous fluorescence-quenching studies have shown that halide ions can associate with cetyltrimethylammonium micellar interfaces and that the Stern-layer ion population may govern the observed quenching response. Accordingly, electrostatic attraction and counterion exchange between bromide and added iodide may increase the local iodide concentration experienced by PSs located near the CTAB interface relative to the analytical bulk concentration. Because the local iodide concentration was not independently determined in the present study, the high *K*_SV_ values observed in CTAB cannot be attributed exclusively to greater PS accessibility or interfacial localization. Rather, they likely reflect a combination of PS distribution, iodide partitioning, counterion competition, and the local micellar environment. Therefore, the CTAB *K*_SV_ values should be regarded as apparent Stern–Volmer constants and compared quantitatively with those obtained in SDS, TX-100, and SB3-14 only with caution.^84,85^

In contrast, the results in SDS micelles indicate that the increase in hydrophobicity effectively promotes a deeper incorporation of the esterified derivatives into the micellar pseudophase. The lower accessibility to iodide ions observed for RBOCT and RBDOD is consistent with their greater affinity for hydrophobic environments, as demonstrated by the log *K*_P_ values. Additionally, electrostatic effects may contribute to this behavior. In the case of the ester derivatives, the reduced net charge and increased hydrophobicity facilitate their partitioning into more hydrophobic and protected micellar regions. Therefore, in SDS micelles, the combined effects of hydrophobic and electrostatic interactions appear to govern the organization of the PSs.

For SB3-14 micelles, all compounds exhibited relatively low *K*_SV_ values, indicating significant protection from iodide. Although RBOCT and RBDOD displayed slightly higher *K*_SV_ values than RB, the differences were considerably smaller than those observed for CTAB and SDS.

The singlet oxygen quantum yields obtained in micellar systems (Table 10) confirm that microheterogeneous environments strongly influence the photodynamic behavior of RB and its ester derivatives. Because RB in homogeneous EtOH was used as an external reference, the values obtained in the micellar systems are reported as apparent singlet oxygen quantum yields (Φ_Δ,app_) and should be interpreted comparatively. The Φ_Δ,app_ values ranged from 0.18 to 0.52, with the highest values observed in SDS. This behavior is consistent with the recovery of fluorescence emission observed upon micellization, indicating that the restoration of monomeric species restores photoactive monomeric species capable of participating in the excited-state processes required for singlet oxygen generation.^10,18,56^

**Table 10:** Kinetic and Photophysical Parameters Obtained Through the Reaction between ^1^O_2_ and DPBF; [PS] = 5.0 × 10^–6^ mol L^–1^; pH 7.4 and 25.0 °C^a^

Surfactant	PS	*k*_DPBF_ (10^–^ ^4^ s^–1^)	*P*_ABS_ (10^–3^W)	*N*_ABS_ (10^–4^E)	γ_Δ_	Φ_Δ,app_
SDS	RB	9.74 ± 0.04	7.10 ± 0.11	1.12 ± 0.02	8.70 ± 0.14	0.52
	RBOCT	9.01 ± 1.25	6.60 ± 0.20	1.05 ± 0.03	8.58 ± 1.22	0.52
	RBDOD	10.40 ± 0.88	8.26 ± 0.07	1.31 ± 0.01	7.94 ± 0.68	0.48
CTAB	RB	5.57 ± 1.20	6.61 ± 0.19	1.05 ± 0.03	5.30 ± 1.15	0.32
	RBOCT	4.56 ± 0.49	6.01 ± 0.11	0.95 ± 0.02	4.80 ± 0.53	0.29
	RBDOD	5.40 ± 0.77	7.61 ± 0.21	1.21 ± 0.03	4.46 ± 0.65	0.27
TX-100	RB	5.82 ± 0.83	6.37 ± 0.64	1.01 ± 0.10	5.76 ± 1.01	0.35
	RBOCT	5.32 ± 0.24	6.11 ± 0.13	0.97 ± 0.02	5.48 ± 0.28	0.33
	RBDOD	4.81 ± 0.28	7.50 ± 0.14	1.19 ± 0.02	4.04 ± 0.25	0.24
SB3-14	RB	3.79 ± 0.35	7.92 ± 0.10	1.25 ± 0.02	3.03 ± 0.28	0.18
	RBOCT	3.63 ± 0.15	7.12 ± 0.26	1.13 ± 0.04	3.21 ± 0.18	0.19
	RBDOD	3.72 ± 0.19	7.77 ± 0.07	1.25 ± 0.03	2.98 ± 0.17	0.18

a RB in a homogeneous medium of EtOH was employed as a standard.^29^

It should be noted, however, that the Φ_Δ_ values determined in micellar media are considerably lower than those obtained in homogeneous solvents. This comparison must be made cautiously because the micellar values are Φ_Δ_,_app_ obtained using RB/EtOH as an external reference and therefore do not represent absolute quantum yields. This apparent reduction may not necessarily reflect a lower intrinsic efficiency of singlet oxygen generation but rather the limitations of the indirect DPBF assay in microheterogeneous systems. Accurate quantification by this method requires that the PS, DPBF, and the generated singlet oxygen be located in close spatial proximity. In micellar media, however, the PS and the probe may partition into different regions of the aggregate, while singlet oxygen has a limited diffusion distance and lifetime. Consequently, the fraction of generated singlet oxygen intercepted by DPBF can depend strongly on probe partitioning and the local micellar environment, affecting the measured response. Therefore, in micellar systems, the DPBF assay should be regarded primarily as a comparative indicator of singlet oxygen generation within the same experimental framework rather than as an absolute quantitative determination of Φ_Δ_.

Overall, the biomimetic micellar systems exhibited a strong affinity for the esterified RB derivatives, effectively modulating their photophysical behavior by reducing aggregation, enhancing fluorescence emission, and preserving their ability to generate singlet oxygen. The comparable Φ_Δ,app_ values obtained for RB, RBOCT, and RBDOD within each surfactant, particularly in SDS and SB3-14, indicate that esterification does not suppress the photosensitizing response after micellar incorporation. Collectively, the results provide valuable insights into the relationship between molecular structure, microenvironment, and photodynamic activity, supporting the potential application of these derivatives as PSs for PDT applications.

## 4 Conclusions

The development of PSs for PDT requires a detailed understanding of how the molecular structure and microenvironment jointly influence aggregation, photophysical behavior, and photoactivity under physiologically relevant conditions. In this context, this work systematically investigated the effects of alkyl esterification on the physicochemical and photodynamic properties of RB by synthesizing and characterizing two ester derivatives, RBOCT and RBDOD, in both homogeneous and microheterogeneous media.

The results show that esterification of the RB carboxylate group increases the lipophilicity while preserving the fundamental electronic characteristics of the xanthene chromophore. In homogeneous media, the derivatives exhibited photophysical properties that were strongly dependent on the aggregation state and the solvent polarity. Increasing hydrophobicity promoted aggregation and fluorescence quenching in aqueous environments, and the alkyl derivatives also showed a reduced level of singlet oxygen generation in water.

The RB series proved to be a useful model for elucidating how hydrophobicity and charge modulate the behavior of xanthene-based PSs in organized systems. Micellar media acted as effective biomimetic platforms, improving solubilization and suppressing aggregation. The combined spectroscopic and physicochemical analyses revealed that dye distribution within the assemblies is governed by a balance among hydrophobic interactions, electrostatic effects, and interfacial hydration.

In micellar biomimetic systems, the investigated microenvironments restored the photophysical profiles characteristic of monomeric species. Among the systems investigated, the most pronounced photophysical recovery was observed for RBDOD in TX-100 micelles, where the fluorescence quantum yield increased by nearly 1 order of magnitude relative to that in aqueous solution, highlighting the strong influence of the local environment on excited-state deactivation pathways. It should be emphasized, however, that TX-100 was employed here as a fundamental nonionic biomimetic model for investigating microenvironment-dependent photophysical behavior rather than as a potential clinical formulation vehicle. In addition, all micellar systems enhanced singlet oxygen generation relative to that of water, indicating that microenvironmental control plays an important role in preserving the photodynamic activity of hydrophobic RB derivatives under physiologically relevant conditions. Overall, the photophysical, photochemical, and physicochemical results provide a comprehensive description of the factors governing the behavior of RB-based PSs in biomimetic environments and support the potential of RB ester derivatives as tunable PSs for PDT applications.

## Supplementary Material



## Notes

Detailed synthesis and characterization of the RBOCT and RBDOD ester derivatives, including ^1^H and ^13^C NMR, FTIR, MS, and quantitative ^1^H NMR (qNMR) purity analysis; normalized FTIR spectra of RB, RBOCT, and RBDOD; dielectric constants and ET(30) values in different solvents; and the protolytic and tautomeric equilibrium scheme of RB (PDF)
