# Supplementary material for: Hydrophobicity Modulation of Rose Bengal Derivatives in Homogeneous and Biomimetic Systems: Implications for Photodynamic Therapy
Source: ACS Omega. 2026 Oct 10;11(41):63595–617. doi: 10.1021/acsomega.6c07700 (PMC13688534; doi:10.1021/acsomega.6c07700)
Supplement: Supplementary file 1 [file ao6c07700_si_001.pdf]

Hydrophobicity Modulation of Rose Bengal Derivatives in Homogeneous and Biomimetic Systems: Implications for Photodynamic Therapy

Luiza Bertoldo Stefanello[a], Yara Shuvisnki Ricken[a], Miguel Sampaio[a], Carlos Vinícius Doerner dos Santos[a], Camila Fabiano de Freitas Marin[a,]

[a] *Departamento de Química, Universidade Federal de Santa Catarina (UFSC), Florianópolis, SC, Brazil*

SUPPORTING INFORMATION

1. Synthesis and Characterization of Rose Bengal Ester Derivatives: Detailed spectroscopic characterization data for Rose Bengal octyl and dodecyl ester derivatives, including NMR, IR and LRMS spectra, are provided below.

Rose Bengal Octyl Ester, Monosodium Salt (RBOCT):

Yield: 72%. **^{1}H NMR (400 MHz, DMSO-d$_6$):** 7.42 (s, ArH, 2H), 3.91-3.93 (t, OCH_2, 2H, J = 6.8 Hz), 1.31-1.20 (m, CH_2, 4H), 1.20-1.19 (m, CH_2, 2H), 1.11-1.04 (m, CH_2, 4H), 1.03-0.97 (m, CH_2, 2H), 0.84 (t, CH_3, J = 7.3 Hz, 3H). **^{13}C NMR (200 MHz, DMSO-d$_6$):** 172.3 (2C, C-3,6), 163.4 (C=O), 157.5 (2C, C-4a,10a), 139.7(C9), 136.6 (2C, C-1,8), 135.4, 134.2, 132.4, 130.4, 129.7, 129.2 (C-1', 2', 3', 4', 5', 6') , 110.7 (2C, C8a-9a), 97.8 (2C, C-4,5), 76.5 (2C, C-2,7), 66.6 (OCH_2, C-1''), 31.8, 29.0 , 28.9, 28.2, 25.5, 22.5 (CH_2, C-2'', 3'', 4'', 5'', 6''), 14.5 (CH_3, C-8'') . **IR (KBr)** 1734 cm^{-1} (ester C=O). **LRMS (EI-)** *m/z* = 1084.53 [M$^-$] (100%). **Purity by qNMR:** 91.5%

Rose Bengal Dodecyl Ester, Monosodium Salt (RBDOD):

Yield: 60%. **^{1}H NMR (400 MHz, DMSO-d$_6$):** 7.48 (s, ArH, 2H), 3.91-3.93 (t, OCH_2, J = 6.3Hz, 2H), 1.28-1.19 (m, CH_2, 12H), 1.12-1.04 (m, CH_2, 4H), 1.04-0.98 (m, CH_2, 2H), 0.92-0.88 (m, CH_2, 2H), 0.85 (t, CH_3, J = 7.2 Hz, 3H). **^{13}C NMR (200 MHz, DMSO-d$_6$):** 172.1 (2C, C-3,6), 163.7 (C=O), 157.5 (2C, C-4a,10a), 139.8 (C9), 136.6 (2C, C-1,8), 135.4, 134.8, 134.2, 132.2, 130.4, 129.2 (C-1', 2', 3', 4', 5', 6'), 111.0 (2C, C8a-9a), 97.7 (2C, C-4,5), 76.4 (2C, C-2,7), 66.6 (OCH_2, C-1''), 31.8, 29.6, 29.5, 29.4, 29.3, 29.2, 29.0, 28.2, 25.5, 22.6 (CH_2, C-2'', 3'', 4'', 5'', 6'', 7'', 8'', 9'', 10'', 11''), 14.5 (CH_3, C-12'').

IR (KBr) 1737 cm^{-1} (ester C=O). **LRMS (EI-)** *m/z* = 1140.61 [M^{-}] (100%). **Purity by qNMR:** 97.1%

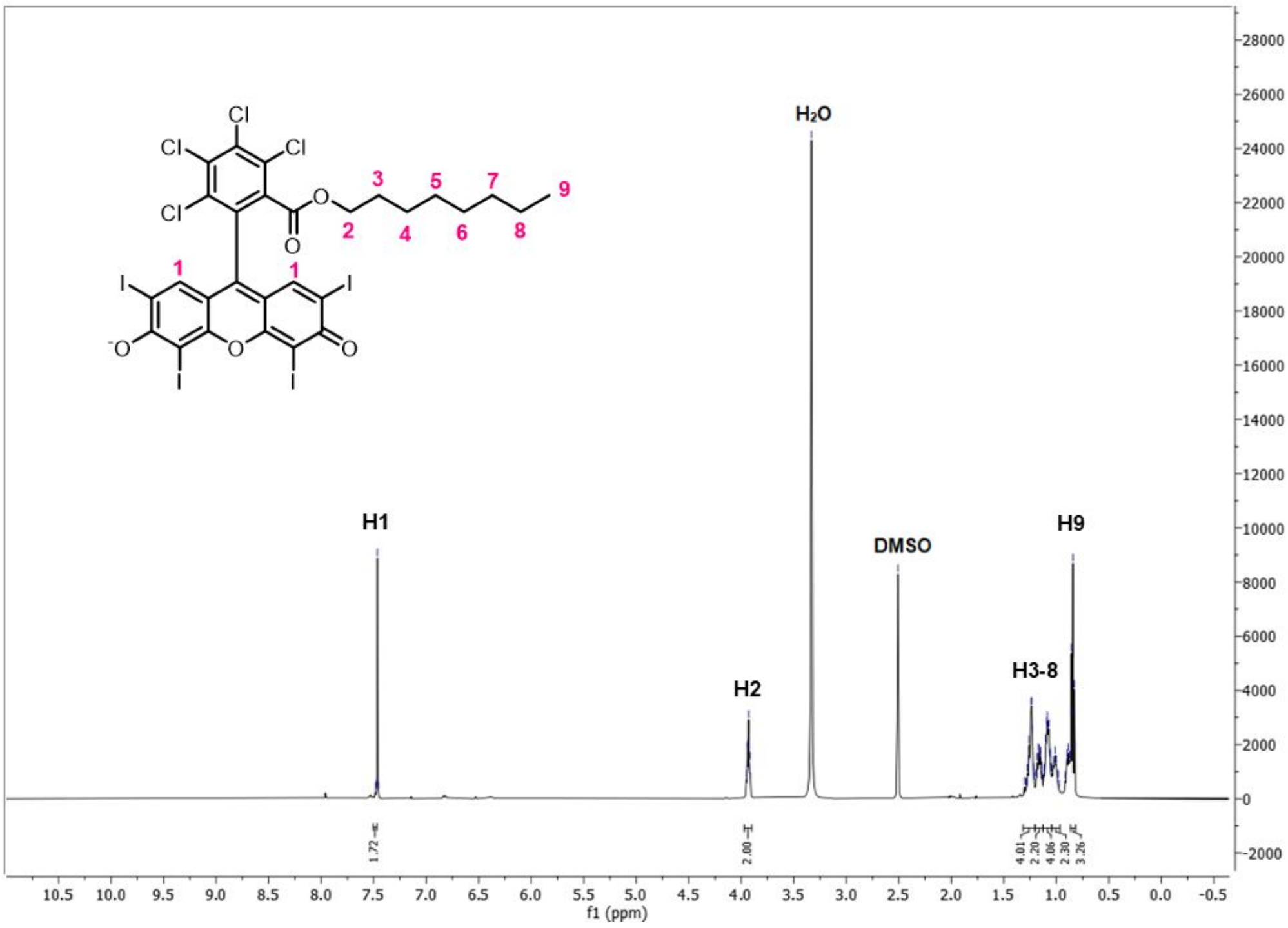

Figure S1. ^{1}H NMR spectrum of RBOCT in DMSO-d$_6$.

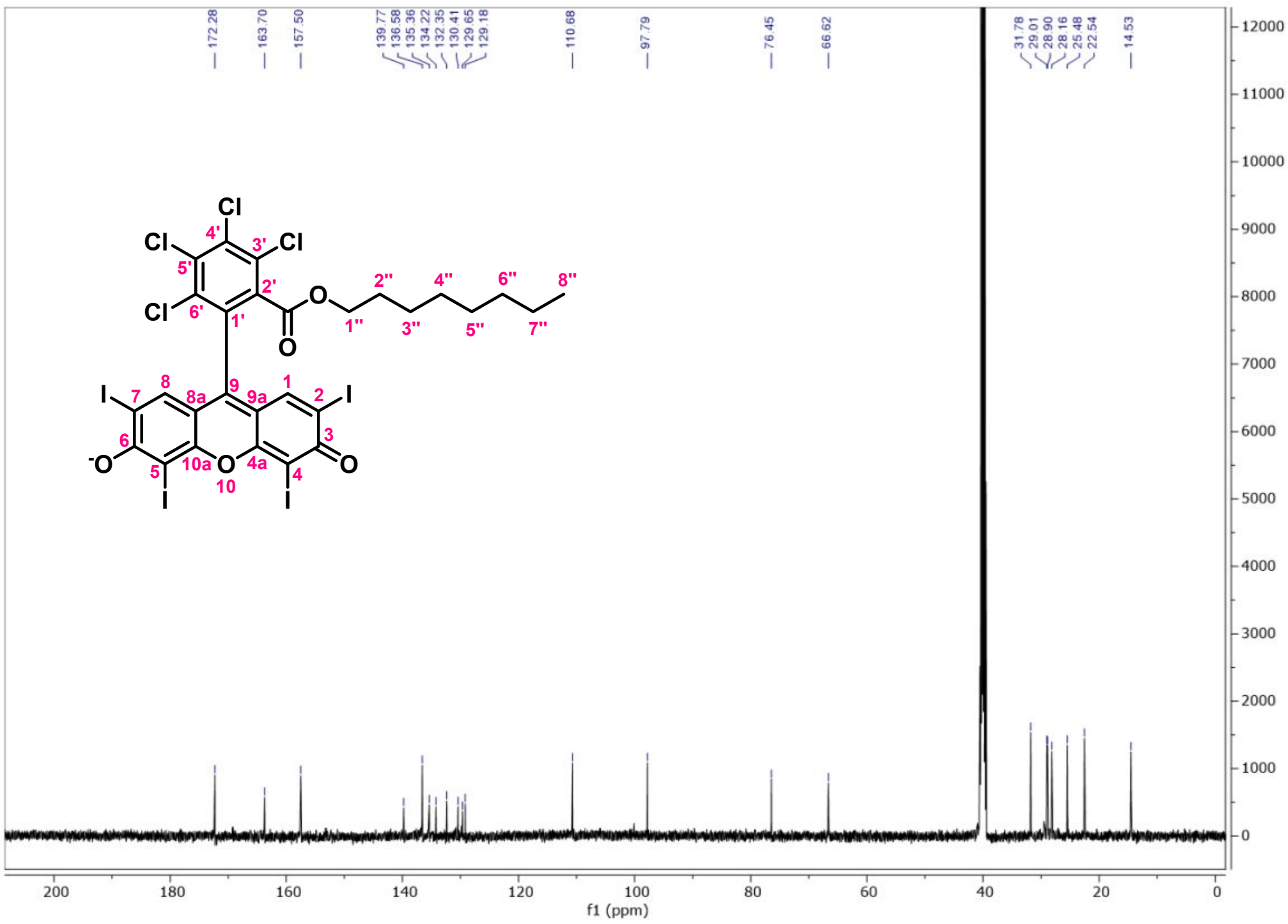

Figure S2. ^{13}C NMR spectrum of RBOCT in DMSO-d$_6$.

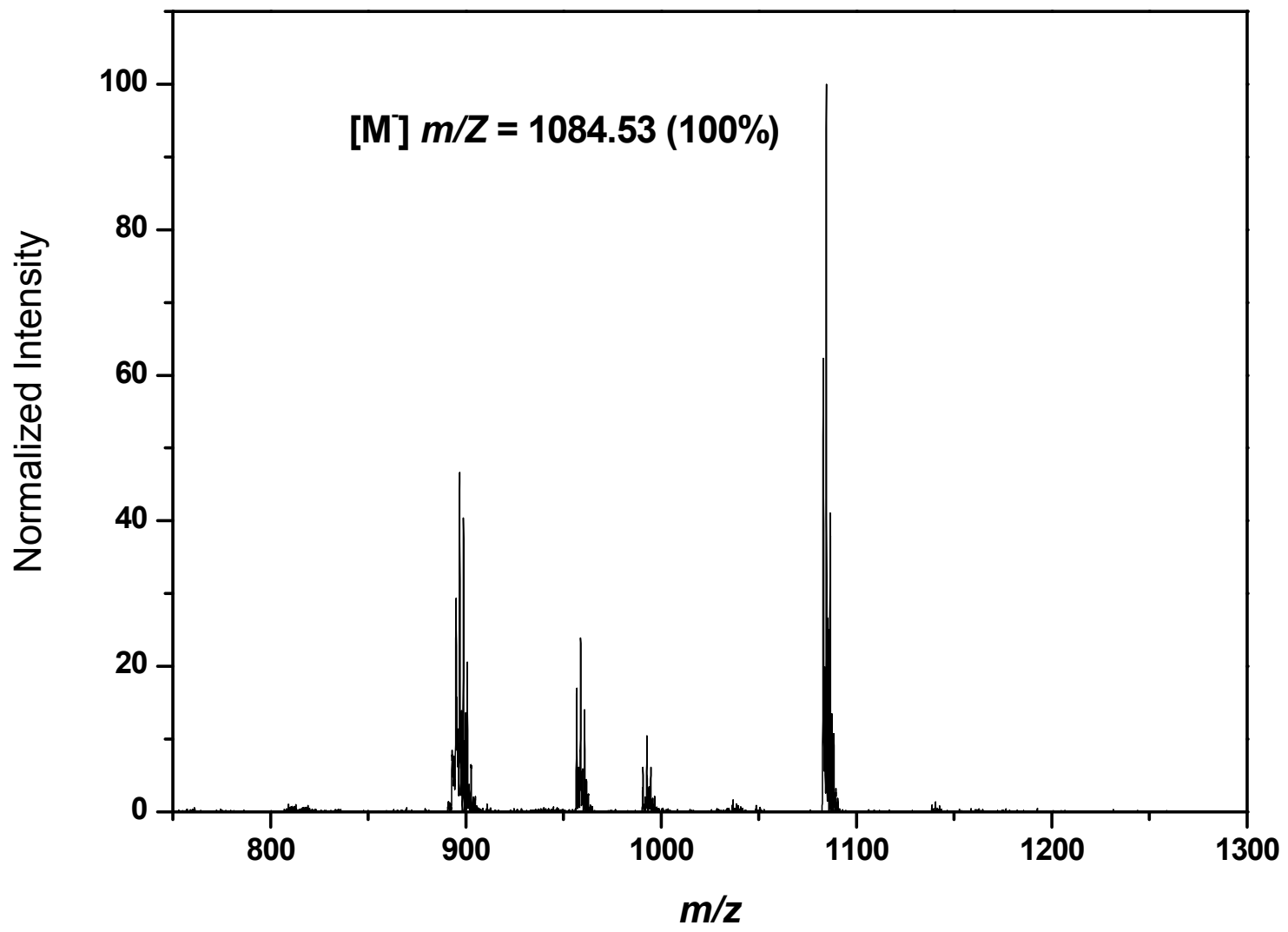

Figure S3. LRMS (EI-, 70 eV) spectrum of RBOCT.

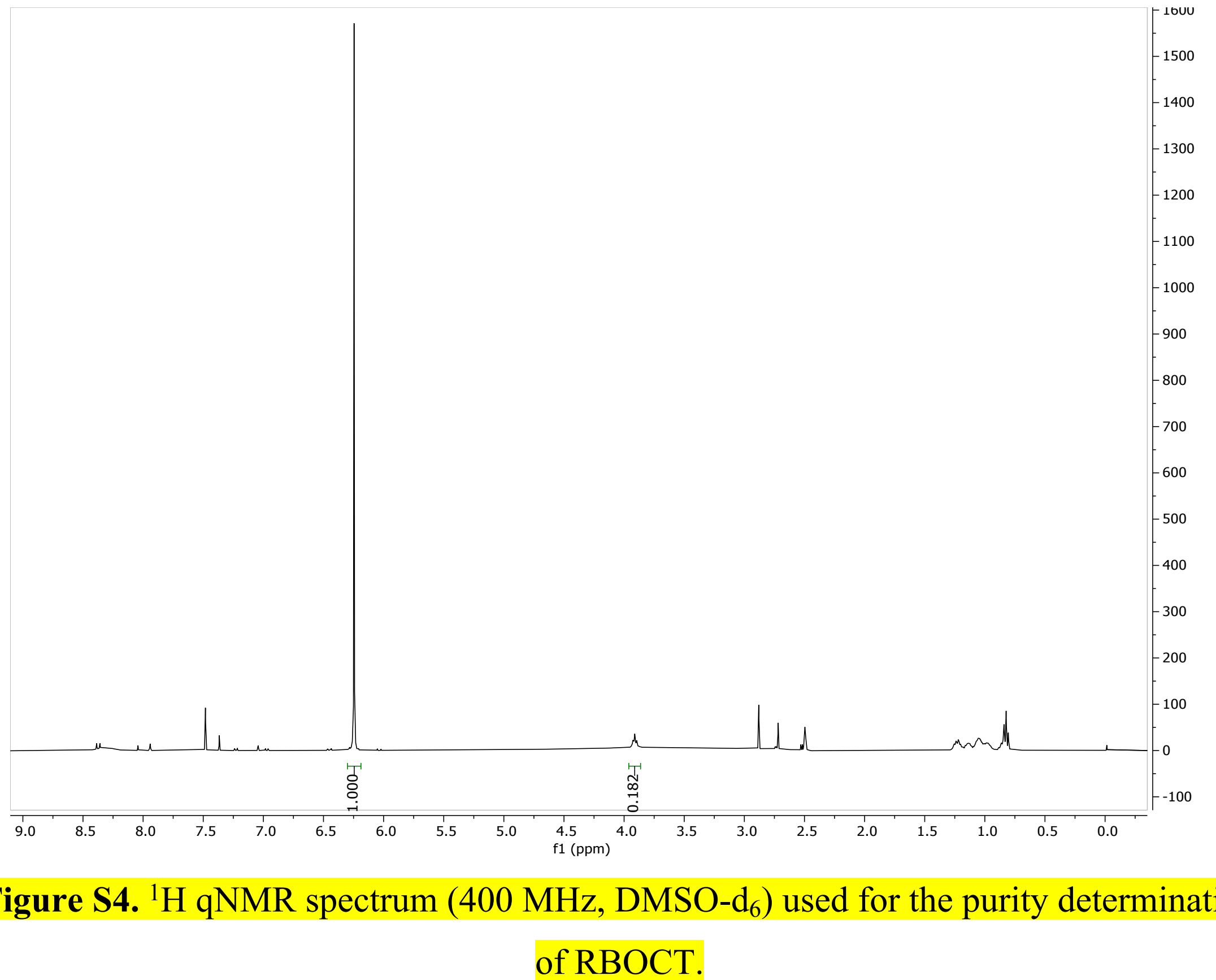

Figure S4. ^{1}H qNMR spectrum (400 MHz, DMSO-d_6) used for the purity determination of RBOCT.

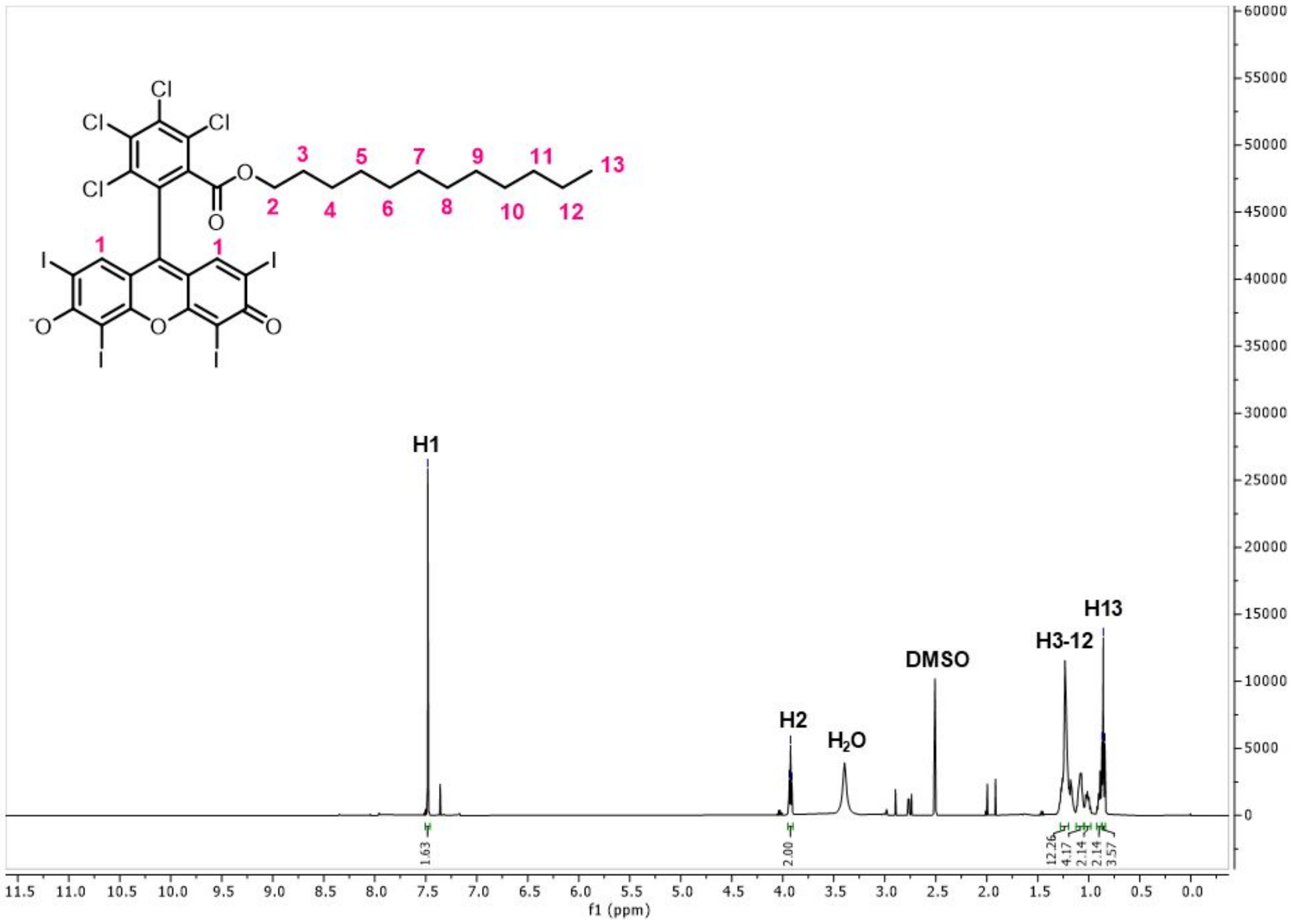

Figure S5. ^{1}H NMR spectrum of RBDOD in DMSO-d$_6$.

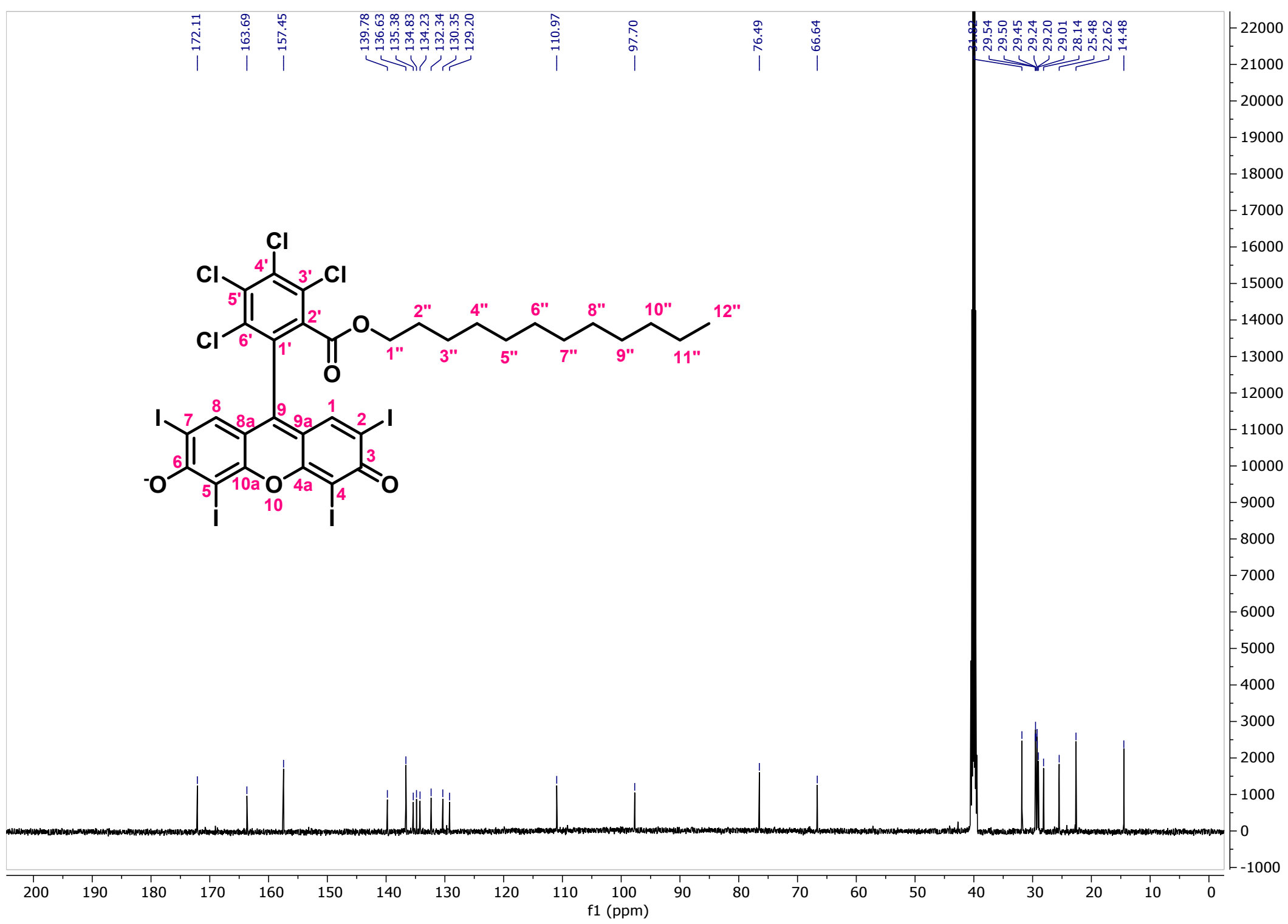

Figure S6. ^{13}C NMR spectrum of RBDOD in DMSO-d$_6$.

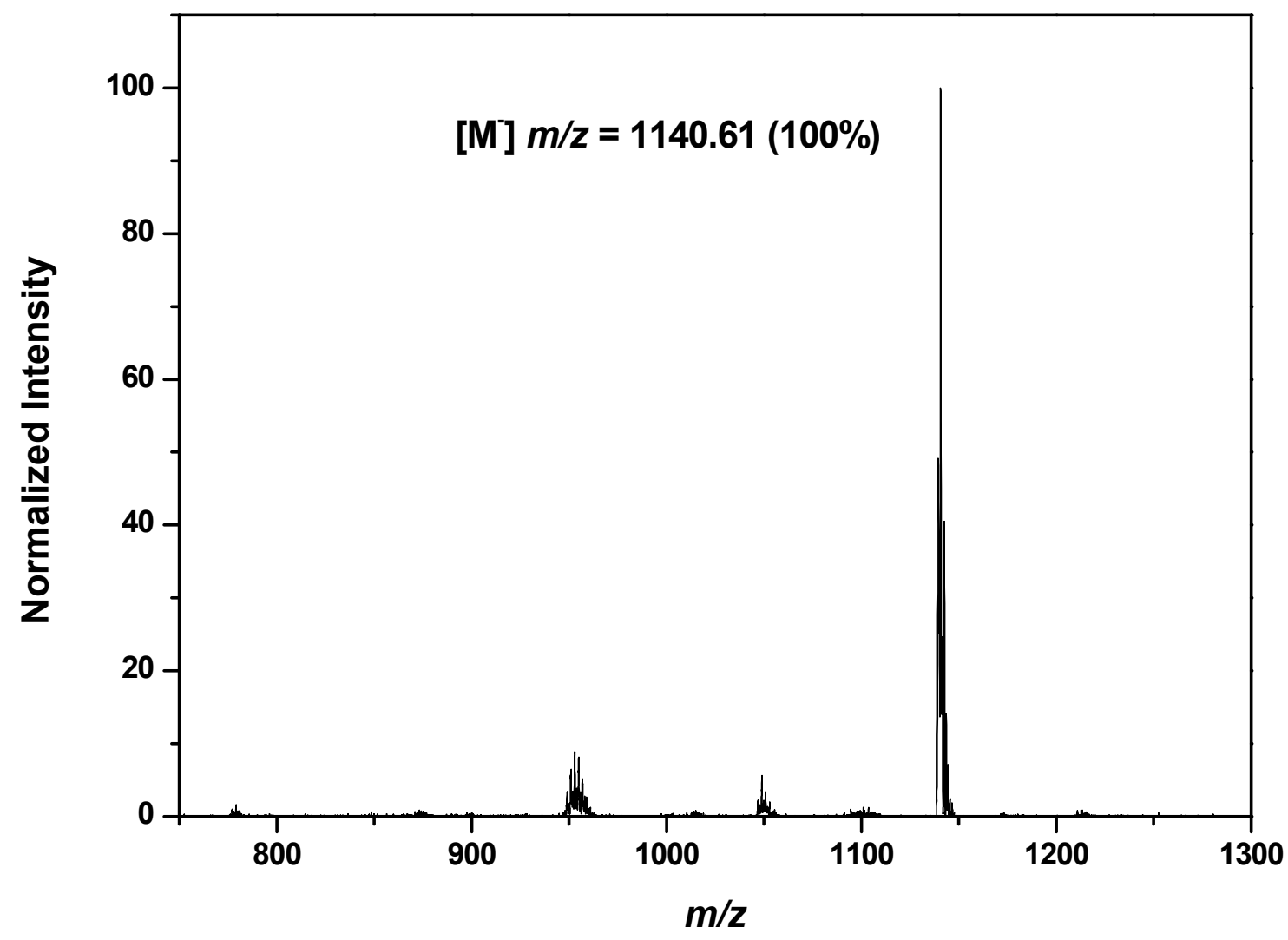

Figure S7. LRMS (EI-, 70 eV) spectrum of RBDOD

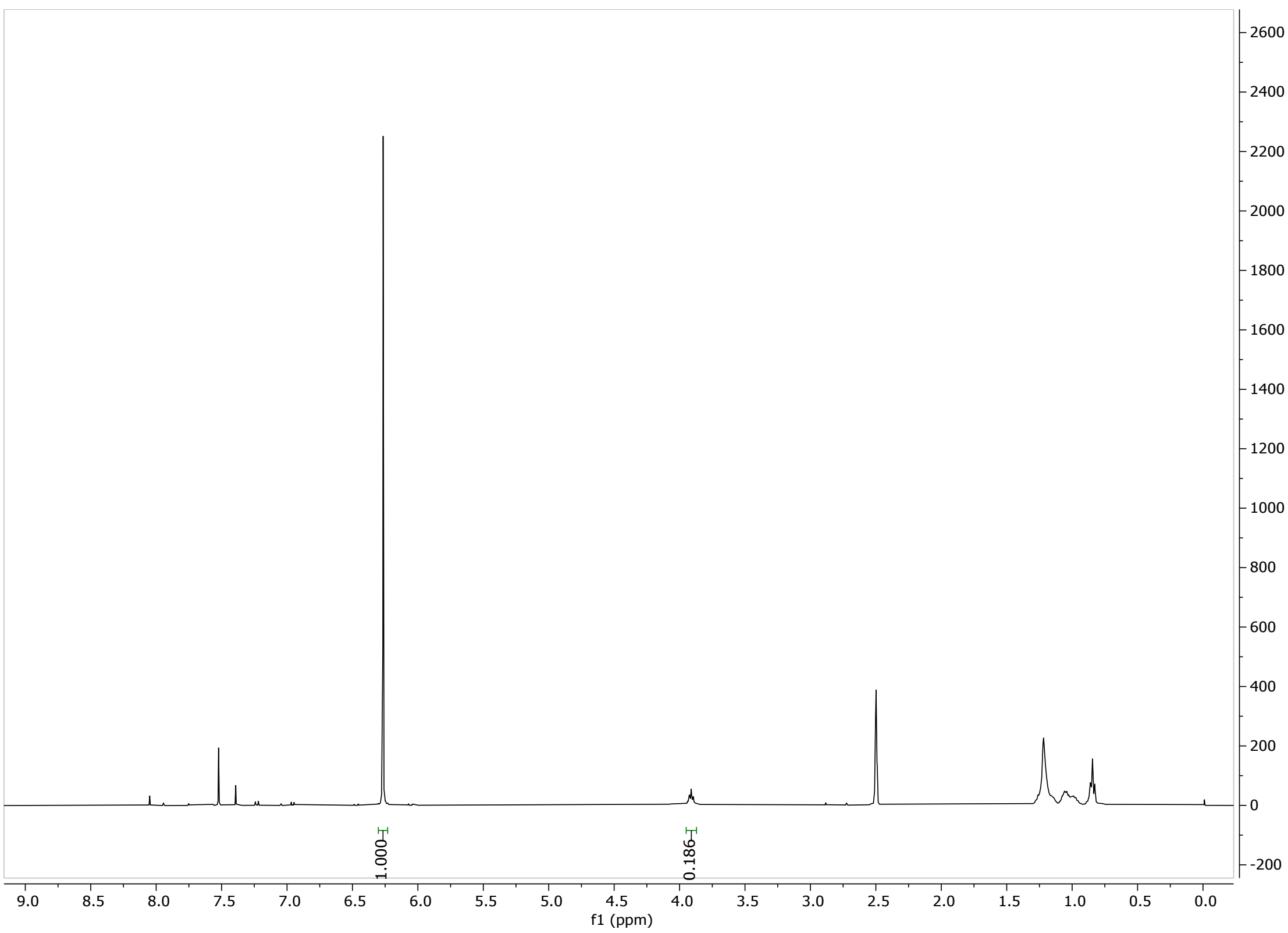

Figure S8. ^{1}H qNMR spectrum (400 MHz, DMSO-d_6) used for the purity determination of RBDOD.

2. Determination of the Purity of Rose Bengal Ester Derivatives: The purities of RBOCT and RBDOD were determined by quantitative ^{1}H NMR spectroscopy (qNMR) using maleic acid as an internal standard. Samples were prepared by accurately weighing the compound and maleic acid directly into NMR tubes, followed by dissolution in DMSO-d_6.

For RBOCT, 10.26 mg of the compound (MW = 1107.86 g mol^{-1}) and 5.46 mg of maleic acid (MW = 116.07 g mol^{-1}, purity > 99%) were used. For RBDOD, 10.72 mg of the compound (MW = 1163.97 g mol^{-1}) and 5.64 mg of maleic acid were used. The samples were dissolved in DMSO-d_6 and analyzed by ^{1}H NMR spectroscopy under quantitative acquisition conditions. The quantitative analysis was based on the integration of the triplets at approximately δ 3.91 ppm, assigned to the OCH_2 protons, relative to the singlet at δ 6.24 ppm corresponding to the non-acidic (olefinic) protons of maleic acid.

The amount of each compound present in the sample was calculated from the ratio of the integrated NMR signals, taking into account the number of protons represented by each signal, the molecular weights of the analyte and internal standard, and the accurately weighed masses.

The purity was calculated according to the following equation:

$$Purity\ (\%) = \frac{I_{analyte}}{I_{IS}}\ x\ \frac{N_{IS}}{N_{analyte}}\ x\ \frac{M_{analyte}}{M_{IS}}\ x\ \frac{m_{IS}}{m_{analyte}}\ x\ P_{IS}$$

I = integrated signal intensity,

N = number of protons associated with the integrated resonance,

M = molecular weight

m = accurately weighed mass

P_{IS} = label purity of the internal standard

For RBOCT, the signal at δ 3.91 ppm showed an integration ratio of 0.182:1 relative to the maleic acid internal standard (Figure S4), corresponding to a purity of 91.5%. For RBDOD, the signal at δ 3.91 ppm showed an integration ratio of 0.186:1 relative to the maleic acid internal standard (Figure S8), corresponding to a purity of 97.1%.

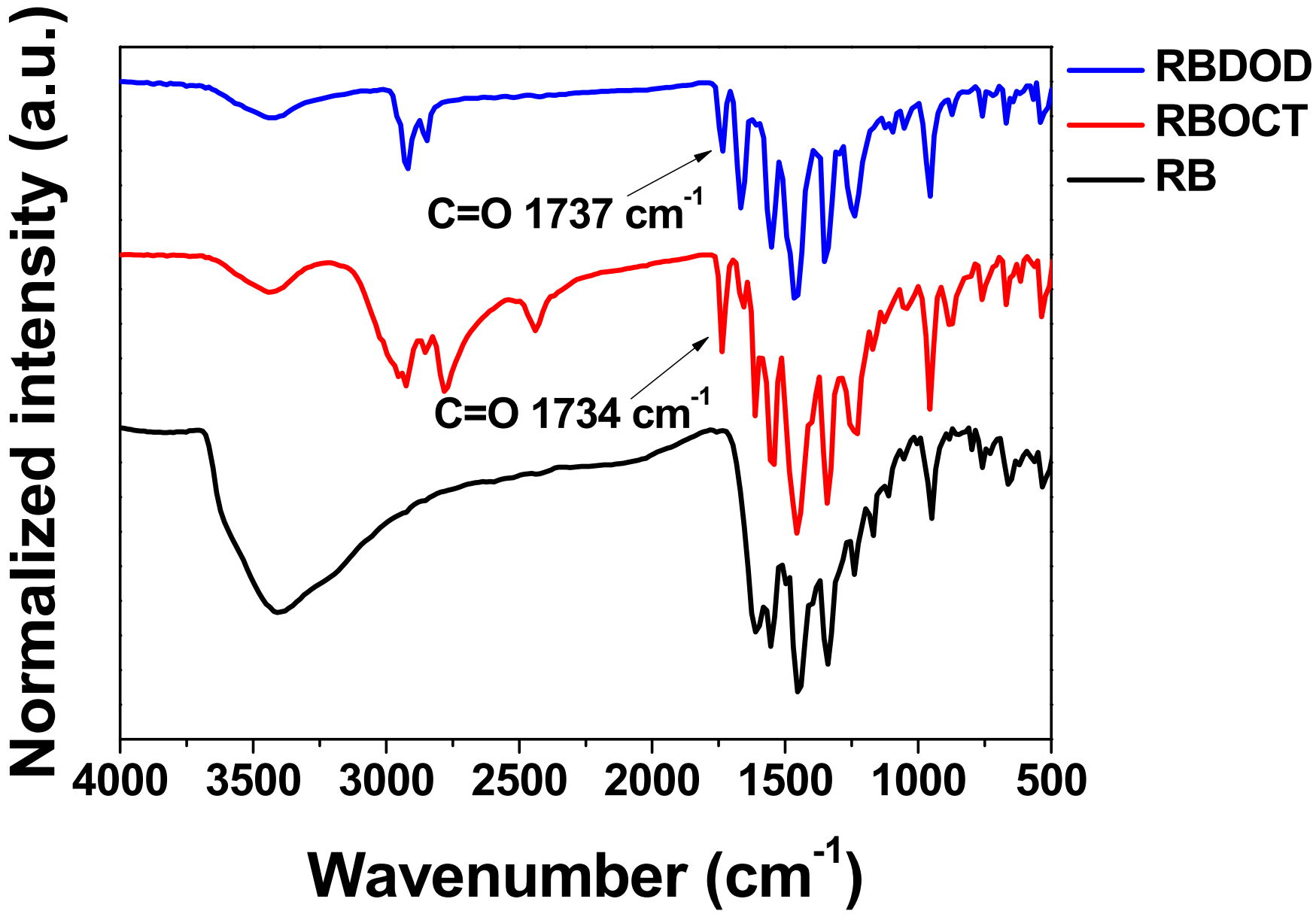

Figure S9. FTIR spectra of RB, RBOCT, and RBDOD recorded as KBr pellets. The spectra were normalized and vertically offset for clarity.

Table S1. Dielectric constants (ε) and $E_T(30)$ values for RB, RBOCT, and RBDOD in various solvents.

Solvent	Dielectric constant (ε)*	$E_T(30)$ RB (kcal mol^{-1})	$E_T(30)$ RBOCT (kcal mol^{-1})	$E_T(30)$ RBDOD (kcal mol^{-1})
Water	80.1	52.75	—	—
MeOH	33.0	51.98	51.33	51.05
EtOH	25.3	51.70	50.87	50.69
PropOH	20.8	51.42	50.69	50.51
ButOH	17.8	51.42	50.69	50.42
PentOH	15.1	51.33	50.60	50.33
OctOH	10.3	—	50.24	50.07
DMSO	47.2	51.05	50.33	50.24
DMF	28.2	51.23	50.42	50.33

* Values extracted from Physical Chemistry Handbook at 293.2 K.

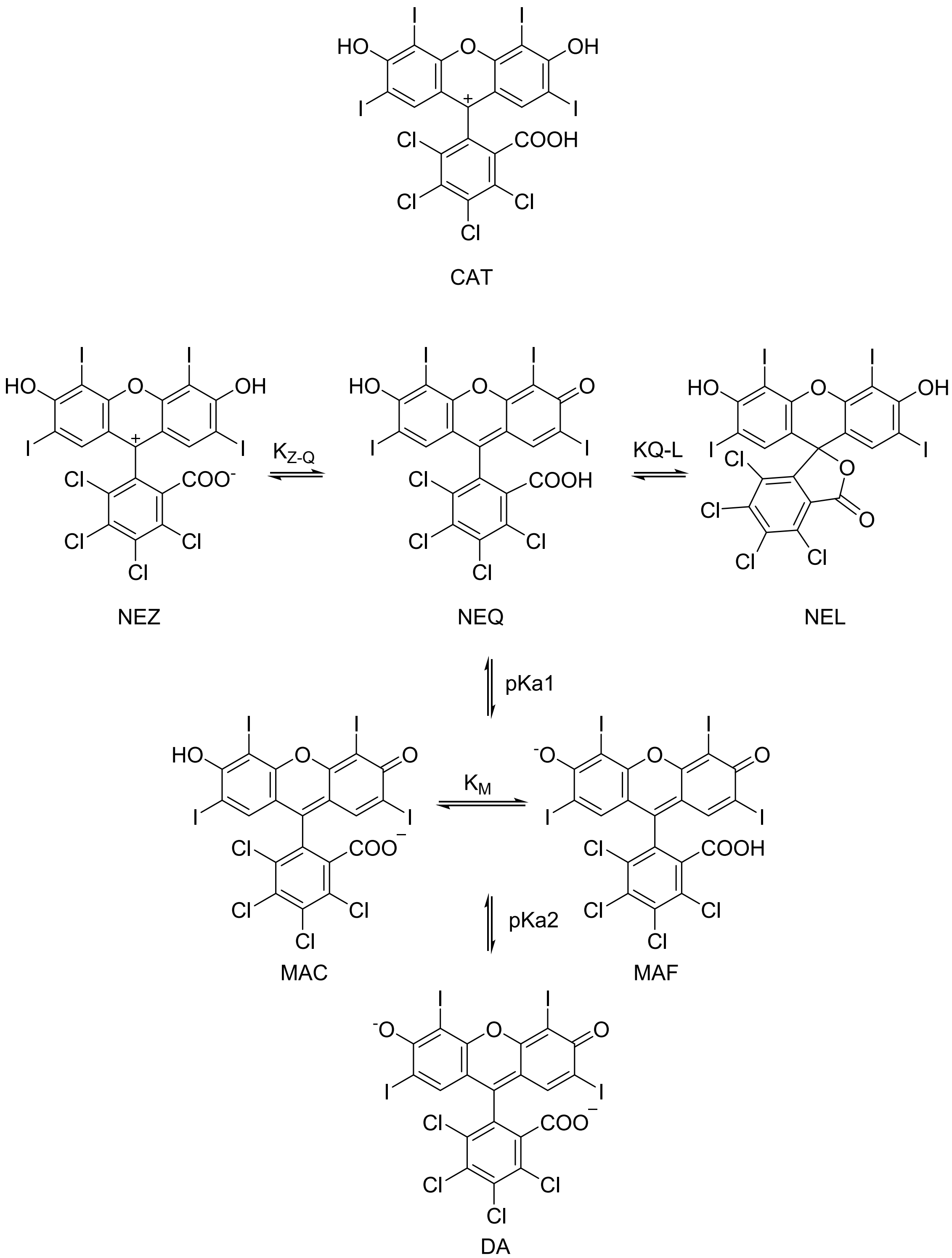

Scheme S1. Protolytic and tautomeric equilibria of Rose Bengal (RB), showing the cationic (CAT), neutral zwitterionic (NEZ), neutral quinoid (NEQ), neutral lactone (NEL), monoanionic carboxylate (MAC), monoanionic phenolate (MAF), and dianionic (DA) species, together with the corresponding equilibrium constants.
